# Introductory Tutorials for Simulating Protein Dynamics with GROMACS

**DOI:** 10.1021/acs.jpcb.4c04901

**Published:** 2024-09-21

**Authors:** Justin A. Lemkul

**Affiliations:** † Department of Biochemistry, Virginia Tech, 111 Engel Hall, 340 West Campus Dr., Blacksburg Virginia 24061, United States; ‡ Center for Drug Discovery, Virginia Tech, 111 Engel Hall, 340 West Campus Dr., Blacksburg Virginia 24061, United States

## Abstract

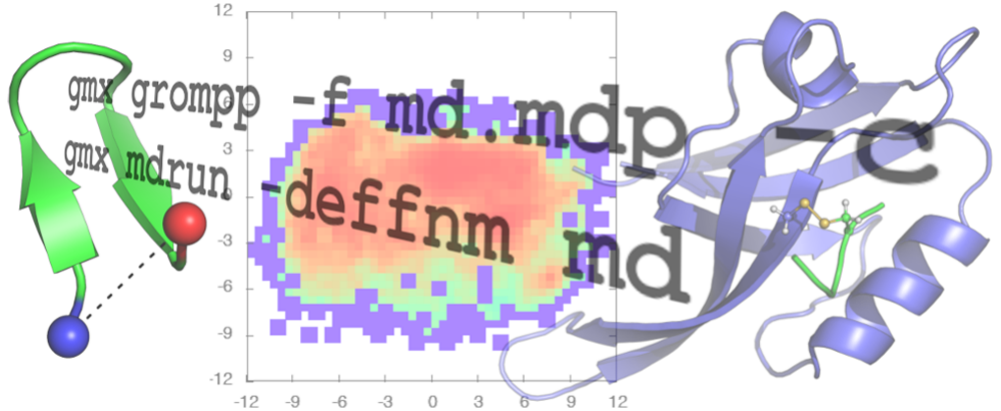

Atomistic molecular dynamics (MD) simulations have become an indispensable tool for investigating the structure, dynamics, and energetics of biomolecules. Continual optimization of software algorithms and hardware has enabled investigators to access biologically relevant time scales in feasible amounts of computing time. Given the widespread use and utility of MD simulations, there is considerable interest in learning essential skills in performing them. Here, we present a set of introductory tutorials for performing MD simulations of proteins in the popular, open-source GROMACS package. Three exercises are detailed, including simulating a single protein, setting up a protein complex, and performing umbrella sampling simulations to model the unfolding of a short polypeptide. Essential features and input settings are illustrated throughout. The purpose of these tutorials is to provide new users with a general understanding of foundational workflows, from which they can design their own simulations.

## Background and Theory

Molecular dynamics (MD) simulations offer the ability to investigate systems of interest with spatial and temporal resolution that exceeds most experimental methods. The accuracy of any MD simulation depends on the quality of the underlying force field, the equation for the energy of a given configuration of atoms and the associated parameters, and the adequacy of sampling. Classical force fields apply principles of Newtonian mechanics to compute the energies of an atomic configuration, from which forces are derived to predict the evolution of the configuration over time.^1^ The issue of sampling is approached in a number of ways, from the “brute force” approach of running extremely long, unbiased simulations, to enhanced sampling methods and external biasing potentials along predetermined degrees of freedom. Therefore, familiarity with advantages and limitations of different force fields,^2^ knowledge of routine simulation protocols, assessing convergence, and experience in enhanced sampling methods are all important for performing robust and scientifically meaningful MD simulations.

Many software packages have been developed to perform MD simulations and analyze their outcomes, including AMBER,^3−5^ CHARMM,^6^ GROMACS,^7−10^ LAMMPS,^11^ NAMD,^12^ OpenMM,^13,14^ and Tinker,^15^ among others. Each of these programs offers different features, force field compatibility, and optimizations for achieving sufficiently fast simulations for systems ranging from thousands to millions of atoms. This tutorial article focuses on guiding the reader through core functions of the GROMACS simulation package in the simulation of polypeptides and proteins.

## Prerequisites and Installation

The exercises described here are designed for use with the current version of GROMACS at the time of writing the article, which is version 2024.1. While there is a general expectation that future versions will retain compatibility and functionality, features and command-line options may change in future versions. GROMACS versioning denotes the year the software was released, with “minor” version changes as a decimal value after the year of the release. Minor versions correspond only to bug fixes in a given release cycle. Thus, it is anticipated that the instructions provided here will be relevant to any version in the 2024 series. Installation instructions for compiling GROMACS on a variety of operating systems and hardware can be found at https://manual.gromacs.org/current/install-guide/index.html. For the purposes of this article, it is assumed that the user has successfully installed an appropriate version of GROMACS. It is also expected that the user is proficient with Unix/Linux file manipulation and navigation, and use of plain-text editors.

These exercises also use the CHARMM36 force field,^16−27^ which is a package that is external to GROMACS. It can be obtained from http://mackerell.umaryland.edu/charmm_ff.shtml#gromacs. Download the most recent version of the force field port,^28^ which as of writing this article was the July 2022 version. All the systems presented here are for proteins in an aqueous environment, therefore the specific version of the force field is CHARMM36m, the most recent version of the protein parameter set.^18^ Future versions of the force field will be released as it is refined and extended, and their availability will be communicated via the GROMACS user forum (https://gromacs.bioexcel.eu/).

To install the CHARMM36 force field, it is a good practice to place the charmm36-jul2022.ff directory in the working directory where the user will be executing all GROMACS commands. It is also possible to install it system-wide, a task that requires administrator privileges.

GROMACS analysis tools produce output files that are plain text and either as raw values or formatted for plotting in the Grace program (https://plasma-gate.weizmann.ac.il/Grace/). Additional analysis described below will be performed with standard Python3 utilities and the Gnuplot program (http://www.gnuplot.info/). Exercise 2 will require the use of PyMOL (https://pymol.org/) to build capping groups and reconstruct missing side chain atoms in the proteins being simulated.

## Exercises

Here, we present three tutorial exercises to introduce the user to core features of GROMACS in preparing, simulating, and analyzing simulations of polypeptides and proteins. The first exercise is the most detailed, providing step-by-step instructions for simulating a protein in an aqueous solution. The second exercise deals with a protein dimer. The third exercise details how to apply an external biasing potential to unfold a small β-hairpin peptide. This article begins with a brief description of GROMACS file types, conventions, and the correct use of the CHARMM36 force field in GROMACS.

### GROMACS File Types

1. .gro: a fixed-format coordinate file with coordinates given in units of nm
2. .pdb: a fixed-format coordinate file used by the Protein Databank with coordinates in units of Å
3. .top: a system topology, defining the complete contents of a system
4. .itp: an “included” topology, defining a specific molecule type, auxiliary parameters, or other topological directives
5. .mdp: “molecular dynamics parameter” file that specifies all relevant settings for performing a calculation or simulation
6. .tpr: a binary run input file that combines coordinates, topology, all associated force field parameters, and all input settings defined in the .mdp file
7. .edr: a binary file containing energy data from the calculation or simulation
8. .xtc: a binary trajectory file in compressed format containing time, box vector, and coordinate information
9. .trr: a high-precision trajectory file containing time, box vector, coordinate, velocity, and force information

### GROMACS Command-Line Conventions

All GROMACS programs are executed as modules of the gmx program, as will be illustrated below. Input and output options are provided as command-line arguments that start with a dash, e.g. -f typically refers to an input file of some sort, -o as an output, *etc*. Some arguments are boolean, e.g. -v for verbose mode and -nov to turn off verbose output. Some arguments require a numerical argument, such as in specifying salt concentration, whereas others take on file names. All GROMACS commands have default values and file names that can be referenced from the manual or by issuing gmx help < program>, where < program> is the name of the command being executed. The full documentation for GROMACS command-line tools can be found at https://manual.gromacs.org/current/user-guide/cmdline.html.

### Notes on Proper Use of the CHARMM36 Force Field

Each force field has certain assumptions inherent in it, which are tied to parametrization methodology and target data, as well as the settings associated with the model physics. It is the latter concern that is worth mentioning here. Shown below are standard settings for using the CHARMM36 force field related to treatment of nonbonded interactions and the use of constraints.

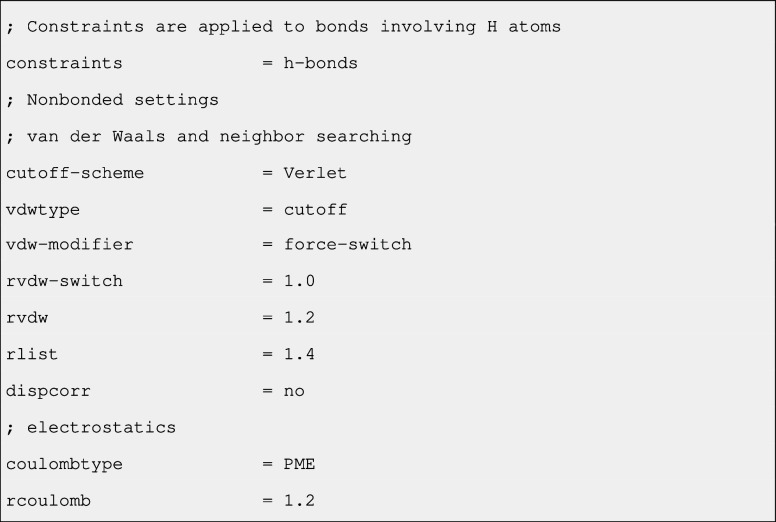



These settings should be considered a part of the force field and not altered unless compelling evidence suggests that alternate values should be used. As such, in this exercise, these settings are applied uniformly in all stages of the protocol. The value of rlist is shown here as the “conventional” value that is used across simulation packages, but with the GROMACS Verlet nonbonded method, this value is automatically tuned to ensure conservation of energy. The value of rlist is only used directly if the user sets verlet-buffer-tolerance = −1.

### Exercise 1: Protein in Water

#### Prepare the Protein Topology

The first system we will construct is a single protein in water with ions. In this case, the protein is ubiquitin, taken from PDB: 1UBQ.^29^ Download the coordinates in PDB format from https://www.rcsb.org/structure/1UBQ. The first step in preparing the system is to define the topology of the protein, set protonation states of side chains and termini, specify disulfide linkages, and build in any missing hydrogen atoms. These processes are carried out in the context of one of the force fields provided with GROMACS, or in our case, downloaded from another source. In classical MD simulations, the bonded structure of a molecule is fixed, therefore there are no protonation or deprotonation events, and cysteine residues will remain in their oxidized (disulfide) or reduced (free thiol) forms. These properties are all defined by the GROMACS tool pdb2gmx. This program prompts the user to make several selections, including choice of force field, treatment of termini, and the water model to be used. Issue the following command:

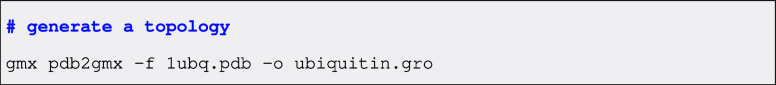



When prompted, choose the CHARMM36 force field, the position of which may vary depending on installation location. In the working directory, it will appear as choice 1, but if in the top-level GROMACS force field directory, it will be choice 9. Enter the corresponding number and Enter to continue.

Next, choose the water model. In the absence of compelling evidence otherwise, the default water model should always be used as it is the water model for which electrostatic parameters are calibrated. The default water model for CHARMM is the TIP3P model,^30^ with modifications to include Lennard-Jones terms on hydrogen atoms.^31,32^ The default water model for each force field in GROMACS is always listed first among the available choices, so choose 1 and press Enter.

In the absence of any other command-line options to specify termini, side chain protonation states, or disulfide linkages, pdb2gmx will use default settings. Assuming that most biomolecular simulations are performed at approximately neutral pH, pdb2gmx will assign charged termini to the N- and C-terminal groups (e.g., −*NH*_3_^+^ and −*COO*^–^) and will assume canonical *pK*_*a*_ values for all amino acids. For ubiquitin, these assumptions are fine but may not be universal for all proteins a user might wish to model. Disulfide bonds are also detected automatically if two cysteine Sγ atoms are within 2 Å, with a distance threshold of 10%, meaning disulfide linkages will be created if these two atoms are within 1.8–2.2 Å.

pdb2gmx will also process any crystallographic water molecules that are present in the input coordinate file. In the case of PDB: 1UBQ, there are 58 water molecules. These water molecules will be added to the topology that is written. Upon completion of pdb2gmx, the user will have the following files in the working directory:

1. ubiquitin.gro: a force field-compliant structure that is protonated according to the assumptions described above.
2. topol.top: the topology of the protein, and in this case, the crystallographic water molecules.
3. posre.itp: an auxiliary topology file that specifies atoms that are restrained by default and their force constants. This file and its use will be discussed in detail later.

#### Define the Periodic Cell and Add Solvent

Globular proteins are typically simulated in an aqueous environment that approximates their natural solution environment. To this end, a volume of space around the protein must be defined and subsequently filled with water, ions, and any other soluble molecules of interest. Because condensed-phase simulations are performed under periodic boundary conditions to prevent edge effects and allow for proper energy conservation, the user must define the size and shape of a central image (also called a “unit cell,” though this term is distinct from its connotation in X-ray crystallography). Here, we will solvate ubiquitin in a rhombic dodecahedral cell. The two GROMACS programs to achieve this task are editconf to define the cell and solvate to add water to the accessible volume around ubiquitin.

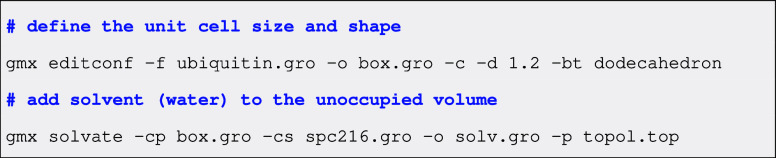



When invoking editconf, the coordinates of ubiquitin are centered within the defined box (with the -c option), and the size of the box is determined from a buffer distance from the maximum and minimum coordinates of the protein; here the buffer is set to 1.2 nm (-d 1.2). While a different box shape like a cube may be more convenient to visualize, the use of a rhombic dodecahedron saves on the required volume of the unit cell and number of water molecules, while still achieving the same periodic distance. The shape (“box type”) is set with -bt dodecahedron.

The solvate program takes the solute coordinates (-cp, historically for “coordinates of the protein”) and uses a pre-equilibrated box of water as the solvent (-cs spc216.gro) and tessellates it through the available volume, deleting any water molecules that overlap with protein atoms. The number of water molecules added to the system is stored and then written to the topology file if the user supplies the -p option, as shown here. It is important to note that the source of solvent coordinates sometimes causes confusion. While in this example, we are topologically representing water with the TIP3P model, the coordinate file is called spc216.gro, because it contains 216 molecules of SPC water.^33^ This coordinate file is reasonable for use with any three-point water model, as the solvent will quickly redistribute upon energy minimization and equilibration to achieve the intrinsic structural and dynamic properties of the chosen water model.

Simulations of proteins in aqueous solution also typically include some amount of salt that is added to the water around the protein. Counterions are often added to offset the net charge of the protein to yield an electrically neutral simulation box. The reason for this convention is that the particle mesh Ewald (PME) method^34,35^ for computing long-range electrostatic forces requires such neutrality, and artifacts may arise if explicit ions are not added.^36,37^ In this case, ubiquitin carries no net charge, so mobile ions may not be strictly necessary. In practice, simulations are often performed to model some experimental or biological conditions, which almost always involve ions.

The genion program requires a binary description of the molecular topology, which is assembled by the GROMACS preprocessor, grompp. The grompp program takes instructions for performing a calculation (energy minimization, MD simulation, *etc*.) and constructs a single input file that contains the coordinates (passed via the -c argument), the topology (specified with -p) and maps the bonded and nonbonded parameters to each of the atoms. Thus, a single input file, with the extension .tpr, contains all the necessary information to carry out a calculation. When adding ions, no calculation is actually performed, but the .tpr file contains all the information that genion needs to determine which molecules are water, such that they can be replaced by ions. By providing the topology via -p to genion, the number of added ions is automatically populated and the number of water molecules is similarly updated. The user also specifies the name of cations (via -pname, for “positive ion name”) and anions (via -nname for “negative ion name”), as well as how many ions are to be added. The latter can be accomplished by explicitly specifying how many of each ion to add, or to allow genion to calculate these quantities based on a desired concentration (via -conc, given in M). Neutralizing ions can automatically be added as part of this concentration with the -neutral option, but it is not necessary here. For this example, we will add 100 mM (0.1 M) NaCl to the system with the following sequence of commands:

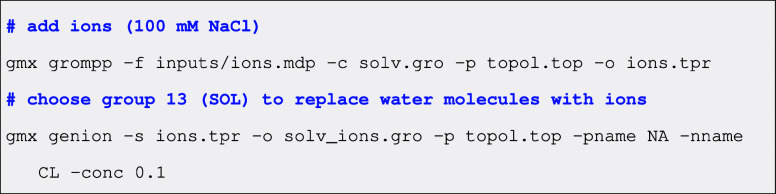



**Figure 1:**
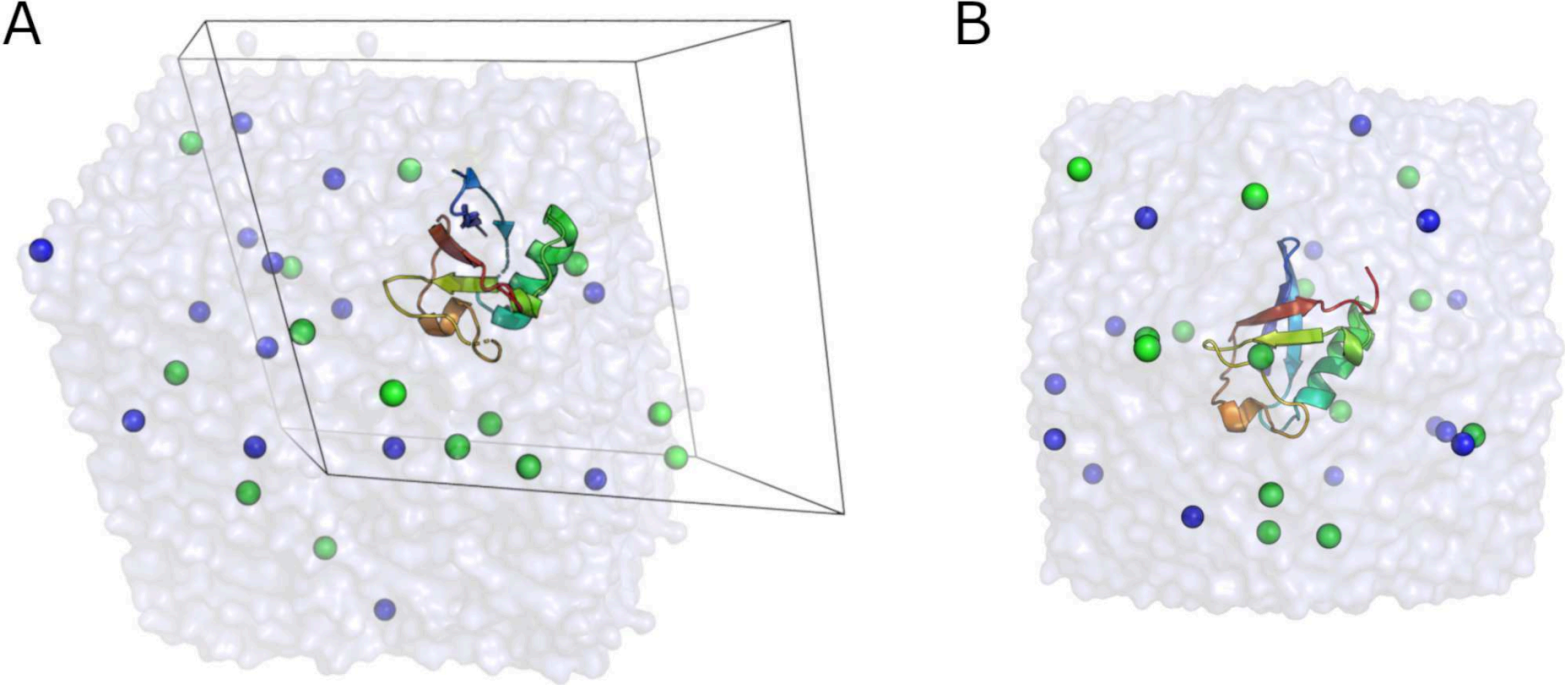
Solvated ubiquitin system, with the protein represented as a cartoon and all water and ions (Na^+^ and Cl^–^ as blue and green spheres, respectively) shown. (A) The default representation with the unit cell shown as a thin black outline. (B) The rewrapped coordinates, showing the rhombic dodecahedral shape.

The constructed system is illustrated in Figure 1A. It is important to note that the shape of the unit cell is wrapped into a triclinic representation and therefore appears misaligned with the rendered box. It is possible to reconstruct the dodecahedral shape in the following way:

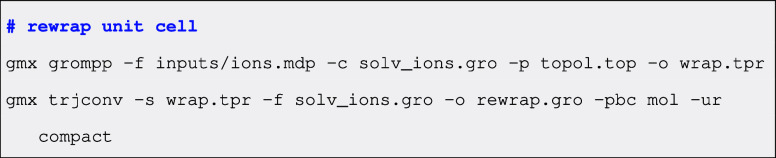



Select 0 (System) for output from trjconv. The result of this operation (Figure 1B) is that the unit cell is rewrapped to yield the true rhombic dodecahedral shape. Note that this process is useful only for visualization; the actual physics in any simulation are unaffected by the representation and for optimal performance, coordinates should not be rewrapped in this way prior to performing any calculation or simulation.

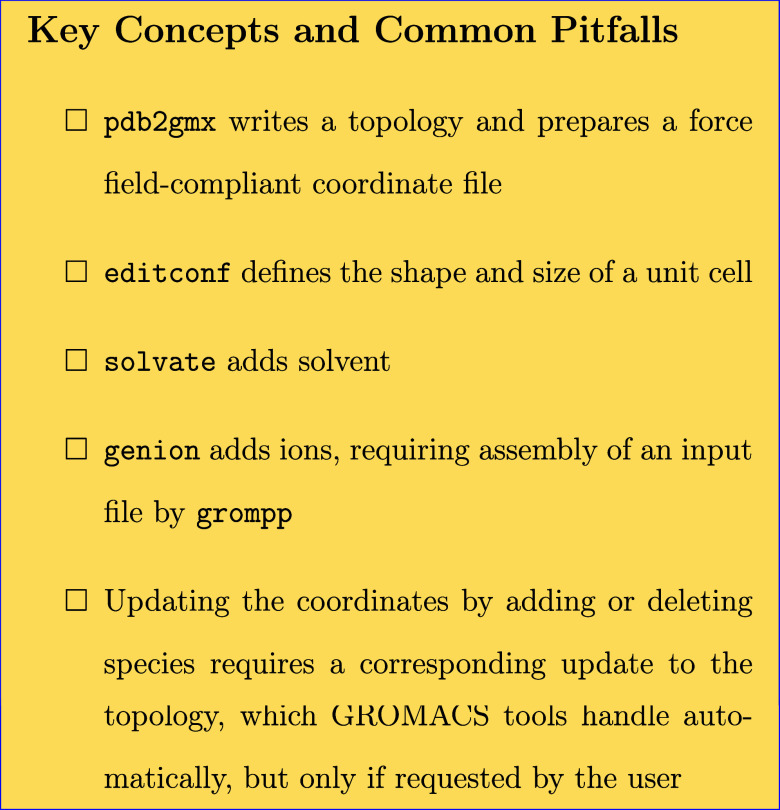



#### Perform Energy Minimization

The process of packing regularly ordered cubic volumes of solvent and subsequently replacing random water molecules with ions may introduce artificiality into the system. Moreover, for a protein structure that has been resolved via X-ray crystallography, it is being exposed to a very different environment than when packed in a lattice. Therefore, bad contacts may exist within the system, as well as suboptimal hydrogen bonding among water molecules and between the water and protein. To resolve these issues, the first molecular mechanics calculation is energy minimization, which moves the atoms along the computed gradient until a threshold maximum force is achieved. Doing so does not guarantee that a system will arrive at a global energy minimum, but it does provide a reasonable starting point for an MD simulation.

We invoke grompp to build the input file, and subsequently execute mdrun to perform the calculation. The mdrun program is the main physics engine in GROMACS and is capable of performing energy minimization, molecular dynamics, and normal modes calculations. As in the generation of a .tpr file for the addition of ions, grompp will take some set of input instructions from an .mdp file; in this case, however, it is important that the settings specify a physically sound model. Aspects such as the nonbonded cutoffs, which are linked to the force field used, must be sensible values. This information is again coupled with the coordinates and topology (passed via -c and -p, respectively) to give the binary .tpr file. This file will now be used by mdrun to perform the energy minimization.

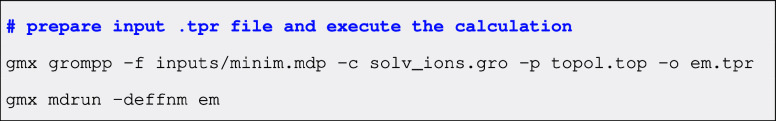



The -deffnm option of mdrun stands for “define file name,” therefore setting a base file name for all input and output files. Rather than individually specifying names for every type of input and output, this approach is very convenient. After running the commands above, one will achieve a result similar to the following, which is printed both to em.log and to the terminal. Coordinates of the energy-minimized structure are printed to the em.gro file.

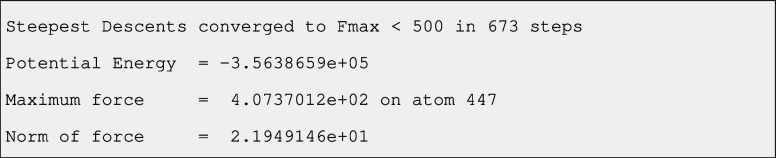



The exact values of potential energy and the number of steps required to converge below the specified force tolerance (here, 500 kJ mol^–1^ nm^–1^) may vary due to some randomness in adding water and the behavior of the compilers used to compile GROMACS.

The potential energy as a function of minimization step can be extracted from a binary energy file (extension .edr) with the energy command, as shown below. When prompted, select 11 (Potential) and 0 (to terminate input).

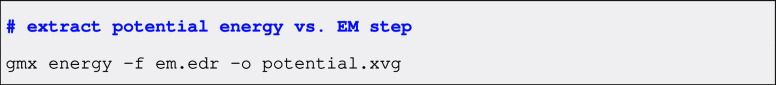



**Figure 2:**
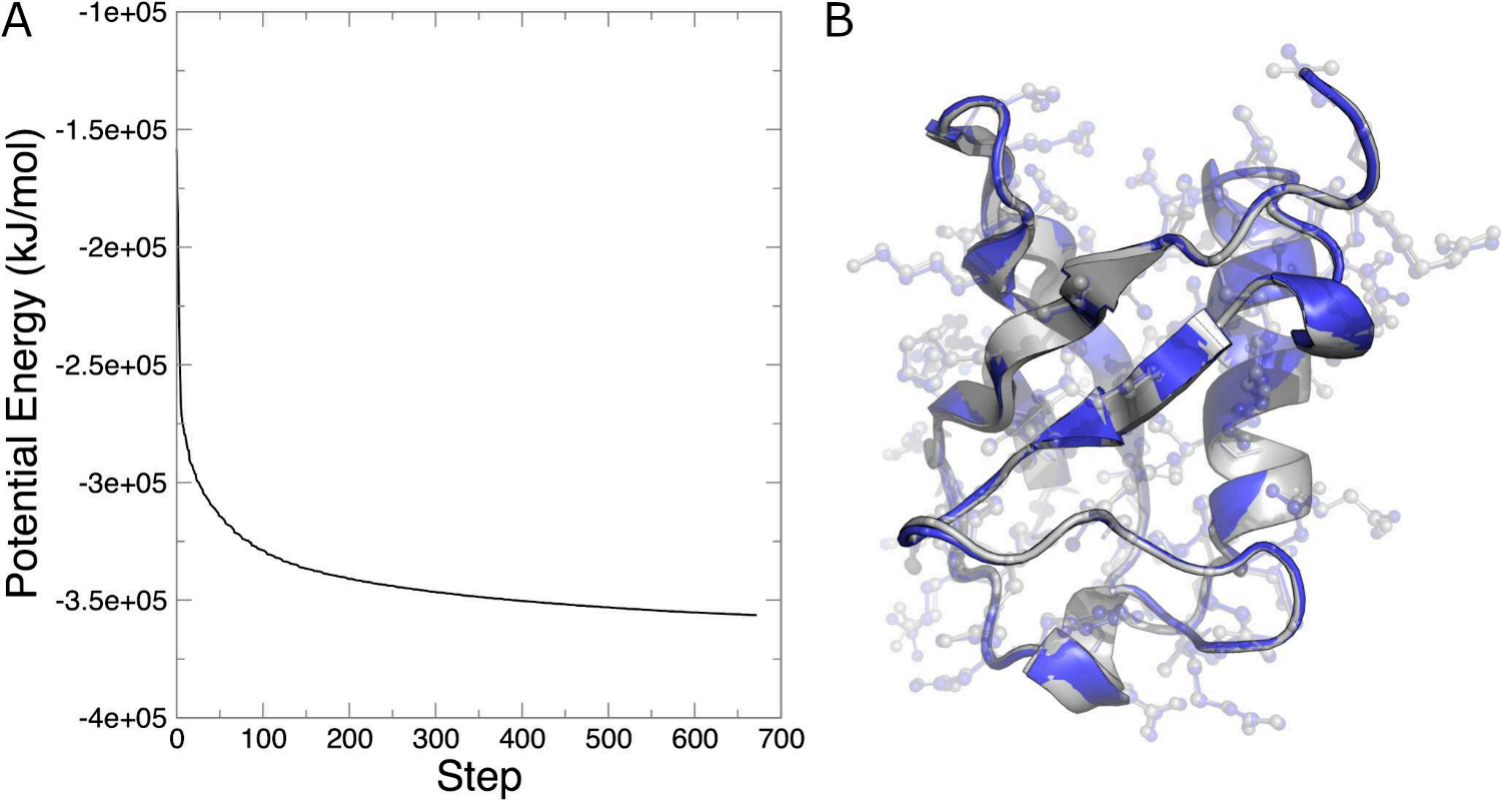
(A) Potential energy as a function of energy minimization step. (B) Overlay of the crystal structure of ubiquitin (gray) and the energy-minimized structure (blue).

Figure 2A shows the progression of the potential energy of the system as a function of energy minimization step. The value drops dramatically in the first few steps as water molecules rearrange to optimize hydrogen bonding. Figure 2B shows an overlay of the crystal structure and the minimized coordinates. There are only subtle changes in the structure of ubiquitin, despite the precipitous drop in the energy of the system. This outcome indicates that minor changes in the protein structure are coupled with much more extensive movements in the water to give a lower energy. As a final note, energy minimization is a nondynamical process. That is, there are no velocities and therefore no sense of kinetics. The atoms move along the gradient to reach a lower-energy configuration.

#### Perform Equilibration

Having reached a suitably low potential energy, the system must now be equilibrated under the desired statistical mechanical ensemble prior to data collection. That is, the thermodynamic variables of interest must stabilize. In this example, we will apply an isothermal–isobaric (*NPT*) ensemble and will equilibrate in two phases, first under a canonical (*NVT*) ensemble, and then *NPT*. During these simulations, it is typical to apply a harmonic restraining force to the non-hydrogen solute atoms. Given that equilibration is initiated from random velocities, there is a chance that unphysical interatomic collisions could distort the structure and produce an unreliable simulation.

It is in this step that we invoke the posre.itp file written by pdb2gmx. This topology file specifies each of the non-hydrogen atoms in ubiquitin and a force constant in the *x*-, *y*-, and *z*-dimensions (the default value for which is 1000 kJ mol^–1^ nm^–2^). The application of restraints is determined by a macro in the topology that follows C preprocessor syntax:

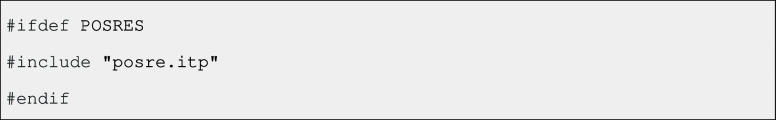



With this construct, the user can selectively choose whether or not to invoke position restraints. When specifying define = -DPOSRES in the .mdp file, the topology macro is satisfied (the POSRES keyword is defined) and the restraint topology is included. When compiling the .tpr file with grompp, an additional coordinate file is provided as input via the -r option. This coordinate file sets the origin of the restraint potential, i.e. where the restraint potential is zero. It is typical that the files specified by -c and -r are identical.

As before, we invoke grompp and mdrun to perform the equilibration steps. First, we perform an *NVT* equilibration to stabilize the temperature in the system.

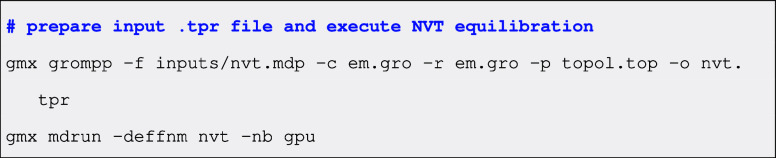



Note that the mdrun command also invokes -nb gpu, which offloads nonbonded calculations to a graphical processing unit (GPU) to accelerate the simulation. Most modern GPUs are supported by GROMACS and can therefore be used to speed up simulations. This 100 ps *NVT* simulation finished in under 30 s using an NVIDIA Titan Xp GPU (a performance of over 300 ns per day).

Extract the temperature over the course of the *NVT* equilibration by again invoking the energy command, selecting 16 and 0 for Temperature and to terminate input, respectively.

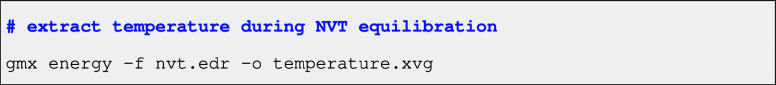



**Figure 3:**
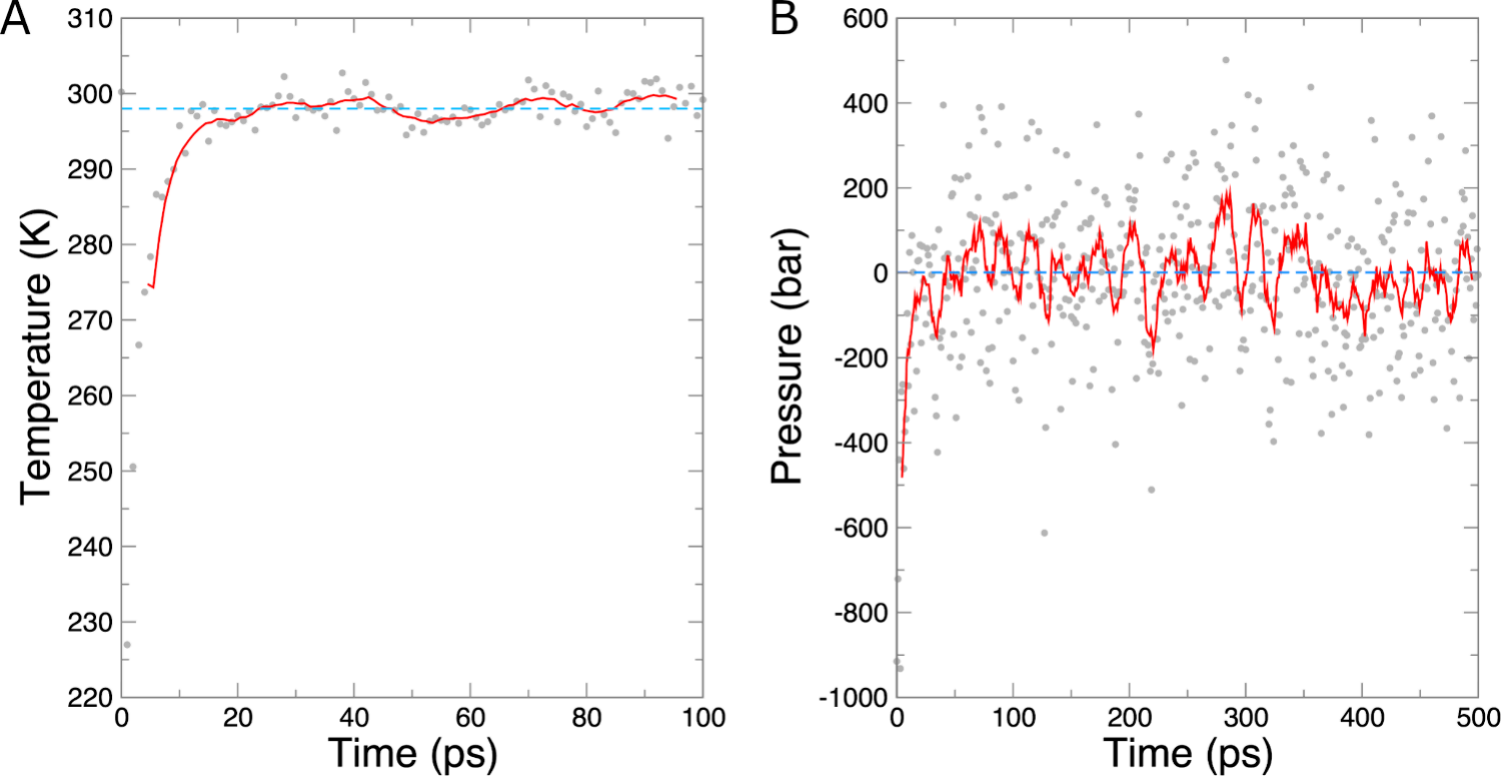
(A) Temperature over time during *NVT* equilibration. Data points are instantaneous values of temperature, the solid red line is a 10-ps running average of the temperature, and the dashed blue line is the target temperature of 298 K. (B) Pressure over time during the *NPT* equilibration. Data points are instantaneous values of pressure, the solid red line is a 10-ps running average of the pressure, and the dashed blue line is the target pressure of 1 bar.

With the thermostat settings given here (the velocity rescaling method of Bussi et al.),^38^ the temperature converged quickly and oscillated around the desired value of 298 K during the final 50 ps of the simulation (Figure 3A).

Proceed to equilibrating the pressure under an *NPT* ensemble in the same manner. Note here that the previous state is passed to grompp via the -t option, which reads a checkpoint file to process state variable information. Using this option ensures an exact continuation between the two equilibration phases, even when changing input settings as we are here.

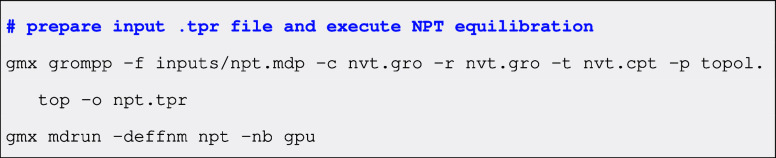



Similar to the *NVT* equilibration, extract the pressure over time with the energy command, this time selecting 17 and 0 for Pressure and to terminate input, respectively. Pressure is a quantity that fluctuates to a much greater extent than temperature, as is clear from Figure 3B. Over 500 ps, an average pressure of −10 ± 189 bar was obtained. Over the final 400 ps of the equilibration, this value was −1 ± 170 bar. Both values are statistically indistinguishable from the target value of 1 bar and therefore suggest adequate equilibration.

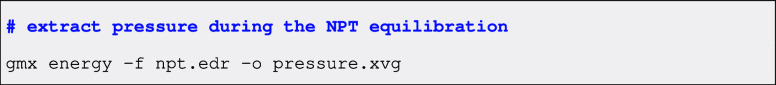



Note that there is no strict requirement for equilibration to be performed in two phases as shown here. It is possible to equilibrate directly under the desired ensemble, however simultaneously generating random velocities and attempting to regulate pressure can sometimes lead to instability. Therefore, it is often beneficial to first stabilize the temperature, followed by the pressure, as is the protocol here.

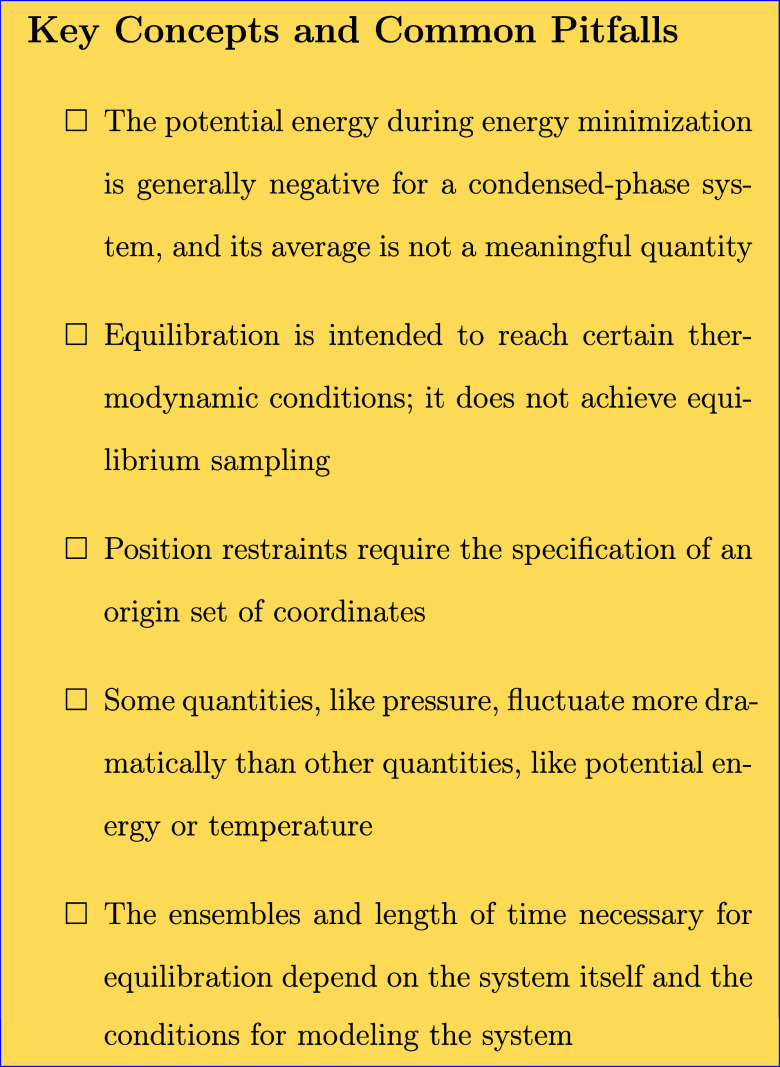



#### Perform an Unbiased MD Simulation

Having sufficiently equilibrated the system, restraints on the protein heavy atoms can now be removed and data collection can begin. This phase of the simulation is sometimes referred to as “production MD” as it is the actual production of usable data. The time frame for the simulation depends on the dynamics of interest; fast motions like side chain rotations may be sampled within a few ns but larger domain motions or folding may require μs–ms of time. Here, we will perform a total of 100 ns of unrestrained MD on ubiquitin in two intervals of 50 ns to illustrate a few key features of GROMACS.

As in previous steps, the input is assembled with grompp, reading in the previous state and producing a .tpr file that is passed to mdrun. Here, the file nomenclature reflects the time interval of the simulation. The input file specifies a simulation length of 50 ns, hence the base of all file names will be md_0_50.

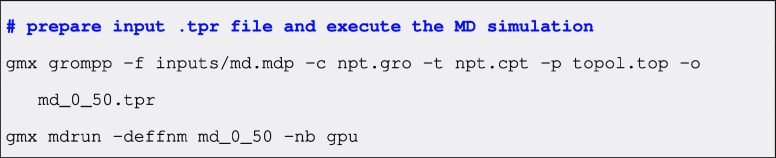



Modern simulations are typically performed for much longer times, and GROMACS offers a convenient mechanism to continue a simulation that has previously completed. Having decided that 50 ns is not enough sampling, we extend the simulation to 100 ns with the convert-tpr program. As input, it takes the previous .tpr file and writes a new one as output that is modified in some way. Here, we change the amount of time specified for the calculation, using -until 100000, meaning the simulation should continue until a time of 100,000 ps (100 ns). It is important to note that while we name the new .tpr file md_50_100.tpr, it actually contains the state at *t* = 0 ns, with instructions to perform a full 100 ns of simulation time.

This naming style is not strictly necessary but is somewhat convenient. By default, mdrun will append to existing output files, whose names are encoded in the checkpoint .cpt files that it writes. If mdrun cannot find these files, it will fail with an error. By specifying separate file names, loading the simulation from a previous checkpoint (-cpi md_0_50.cpt, corresponding to *t* = 50 ns), and telling mdrun to not append to any files (via -noappend), we will obtain output files with the base name of md_50_100 that actually correspond only to that time interval. Execute the second half of the simulation as follows:

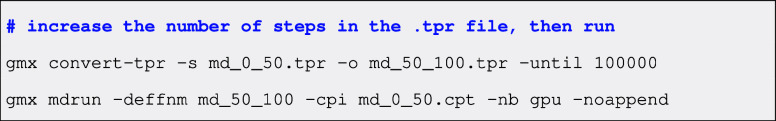



#### Analyze the Simulation

The first step in analyzing the simulation outcome is assembling and reimaging the trajectory so that it can be conveniently visualized. That is, we need to concatenate the two .xtc files into one continuous file, and subsequently account for periodic boundary effects. These effects include molecules “jumping” from one edge of the central image to another and molecules appearing “broken.”

To concatenate trajectory files, use the GROMACS utility trjcat. It accepts any number of trajectory files as input (to the -f option) and outputs a single trajectory file after removing duplicate frames (the default behavior, which can be changed with the -cat option). Following concatenation, periodic boundary effects are removed with the trjconv program by putting all of the molecules in the central image (with -pbc mol, wrapping the unit cell into a compact shape (here, a rhombic dodecahedron) with -ur compact, and centering a desired selection in the unit cell (with -center). Note that for more complex systems, additional steps may be necessary to properly reimage the system, but for a system of a protein (or any single solute) in water, this step should solve any imaging issues. After reimaging the system, the solute (here, ubiquitin) may still be rotating around in the center of the unit cell, so applying a fitting algorithm is useful for visualization convenience. Again, we invoke trjconv on the reimaged trajectory, this time fitting to a reference structure (contained in the md_0_50.tpr file, e.g. the equilibrated coordinates) to remove global rotational or translational motion (with -fit rot+trans). The first invocation of trjconv centers on the protein (by selecting group 1, Protein) and outputs the entire system (group 0, System). The second trjconv step retains only ubiquitin, stripping out all the water and ions because they are not relevant to the analysis demonstrated here. For fitting, global rotation and translation are removed by fitting to the backbone (group 4), eliminating the influence of high-frequency movements of side chains, and writing out only the protein coordinates (group 1).

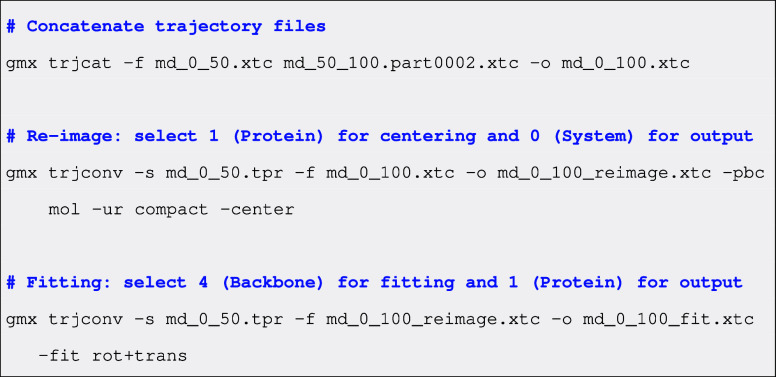



Given that the reimaged, fit trajectory now only contains protein atoms, it is convenient to generate matching reference coordinate and .tpr files. To do so, we perform a similar trjconv operation on npt.gro, which holds the coordinates at *t* = 0 ns, and we use convert-tpr to extract the protein coordinate and topology information from the existing .tpr file. These files can be used for subsequent visualization and analysis.

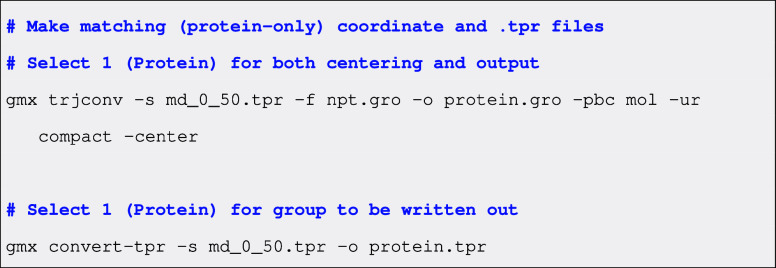



Visual inspection of the trajectory should always be the first step in analyzing any simulation, to understand what happened over the course of the trajectory. What one observes at this stage may motivate future analysis. Here, we present some general analysis methods that are commonly employed, but the user should always have a plan for analysis that will answer the scientific question(s) of interest.

**Figure 4:**
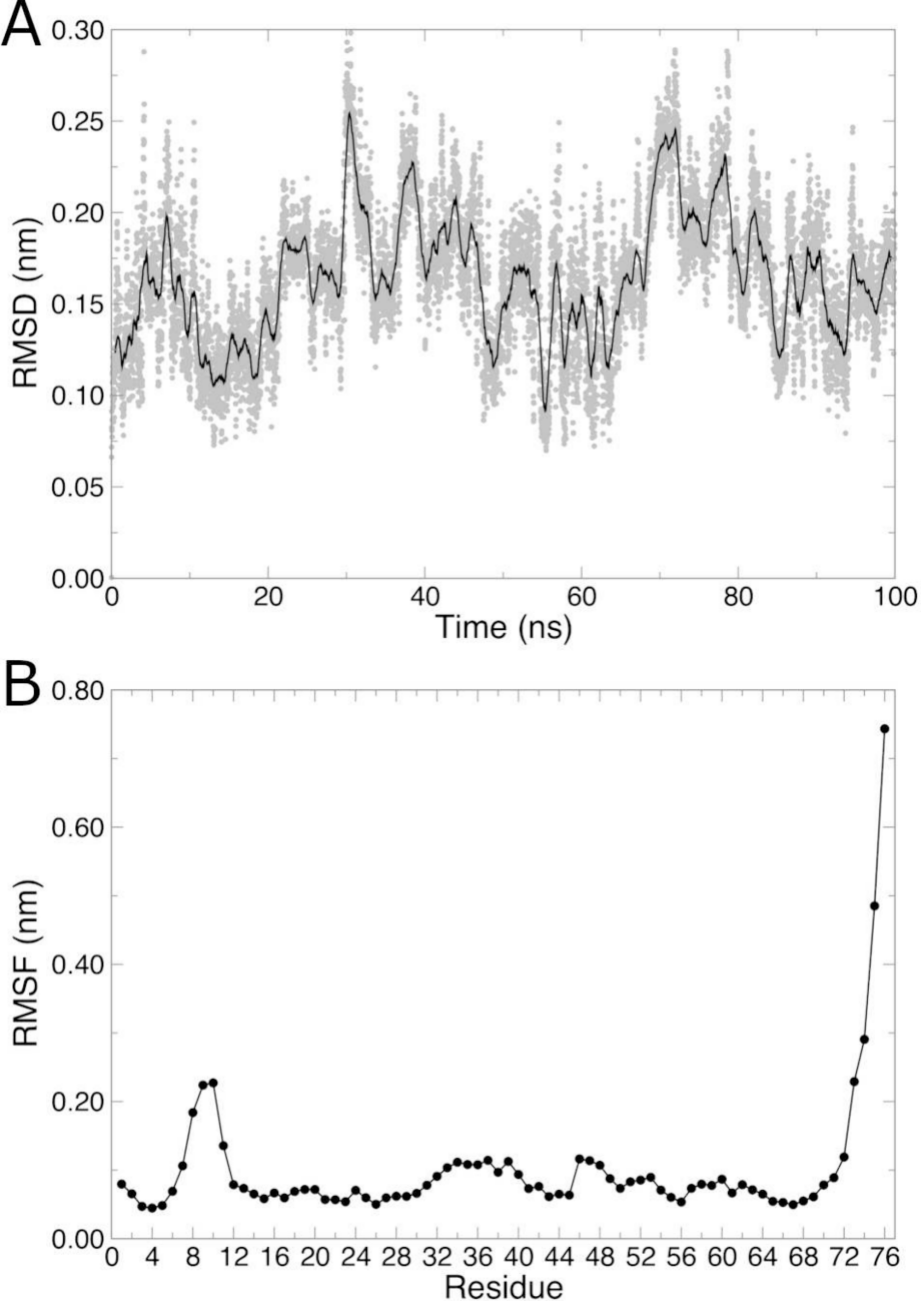
(A) Backbone RMSD over time. Data points are instantaneous values of RMSD, and the solid black line is a 1-ns running average. (B) Cα RMSF for each residue.

One may first want to assess how much the structure of the protein changed over time, which is conventionally calculated with root-mean-square deviation (RMSD) against some reference structure (e.g., the equilibrated coordinates or the crystal structure). The rms program in GROMACS performs this calculation. Choose group 4 (Backbone) for both fitting and calculation groups. Typically, RMSD does not include side chain atoms, which are often very flexible and whose contributions to RMSD are not necessarily indicative of any real change in the protein structure. Analyzing the backbone RMSD is a direct way of quantifying any deviations in secondary and tertiary structure of the protein. Results of this calculation are shown in Figure 4A.

Similarly, the flexibility of the protein can be assessed using root-mean-square fluctuation (RMSF) calculations, with the rmsf program. Here, we will compute the RMSF of Cα atoms (group 3 when prompted), and printing the output per residue (rather than by atom number) with the -res option. Formally, the -res option causes the atoms of each residue to be averaged to give a single RMSF value per residue, but since we are only choosing one atom per residue, the average is the same as the actual value of the RMSF for that atom, and the output is more conveniently interpreted as residue number, rather than atom number (Figure 4B).

From the RMSD analysis, the protein structure changed very little over time, sampling values between 0.1–0.2 nm (1–2 Å, Figure 4A). Much of that deviation is likely attributable to the very flexible C-terminal residues (Figure 4B). While RMSF formally quantifies the oscillation of coordinates about an average position, residues with high RMSF values often indicate areas of structural change throughout the simulation. Visualization of the trajectory confirms that the last few residues in ubiquitin are highly mobile and deviate from the reference coordinates, but the protein otherwise undergoes very little conformational change, as expected.

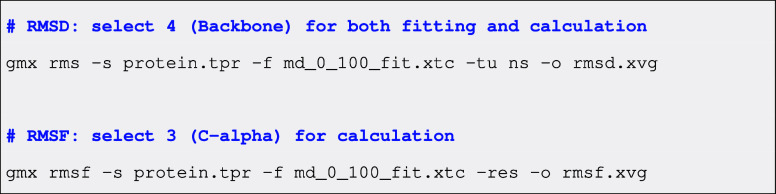



**Figure 5:**
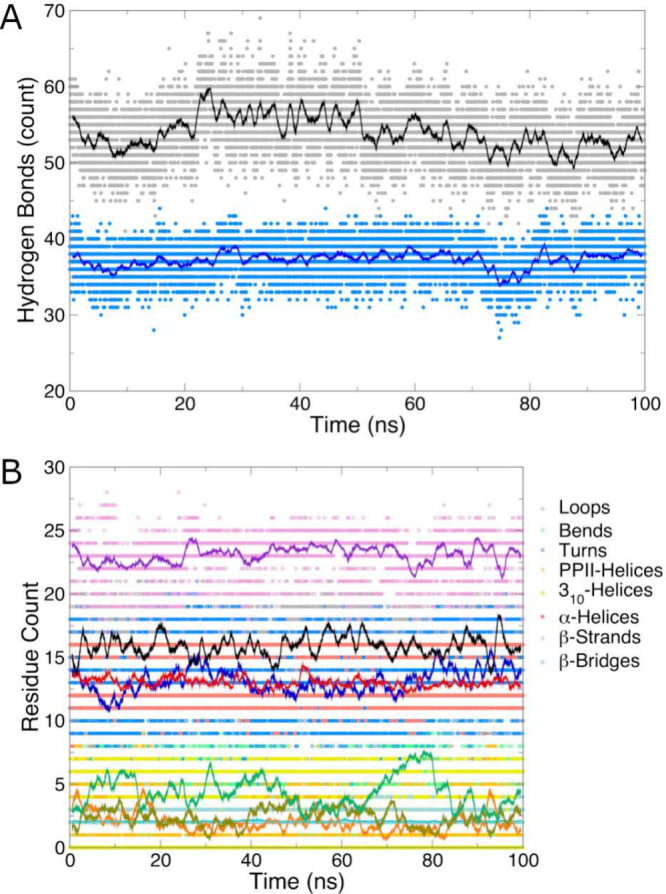
(A) Number of hydrogen bonds over time for all protein atoms and the backbone. (B) Number of residues in each secondary structure type, as assigned by DSSP. For both plots, data points are instantaneous values, and the solid lines are 1-ns running averages. No π-helices were detected in the trajectory, so this time series was omitted for clarity.

Assessing protein structure during an MD simulation often includes characterization of its secondary and tertiary structures. Counting hydrogen bonds can provide insight into both aspects of protein structure. The hbond program in GROMACS counts hydrogen bonds based on geometric criteria (donor–acceptor distance ≤3.5 Å and hydrogen-donor–acceptor angle ≤30° by default). The hbond program prompts the user for two selections of atoms containing donors and/or acceptors; these groups must be exactly identical or completely nonoverlapping. Here, we will analyze the total number of hydrogen bonds in ubiquitin and those involving just the backbone. It is important to note the nomenclature that GROMACS uses for the polypeptide backbone. The “Backbone” group contains only N, Cα, and C atoms. The “MainChain” group includes all of the Backbone atoms but also includes the O atoms. Lastly, the “MainChain+H” group contains the MainChain atoms as well as the amide H atoms. Given that hydrogen bonds are determined explicitly through the use of an angle involving hydrogen atoms, computing “backbone” hydrogen bonds actually requires the use of the “MainChain+H” group, not “Backbone.” We will also analyze all hydrogen bonds in ubiquitin over time by issuing the following two commands. Results of these calculations are shown in Figure 5A.

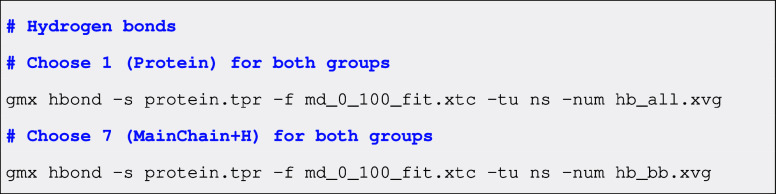



Recent versions of GROMACS implement the DSSP algorithm^39^ for secondary structure assignment in the dssp program.^40^ This algorithm assigns secondary structure elements in proteins based on hydrogen-bonding patterns, providing a time evolution of secondary structure throughout a trajectory, as shown in Figure 5B. Given the nature of secondary structure assignment, the group analyzed must also be MainChain+H as was the case for analyzing backbone hydrogen bonds. Issuing the following command will analyze secondary structure in the trajectory.

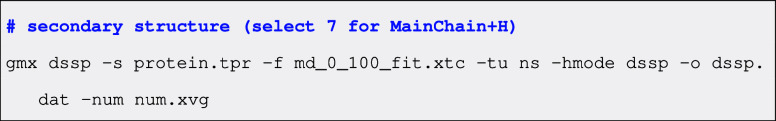



The GROMACS program analyze can be beneficial to compute averages and error estimates for time series data. Calculate the average number of residues of each type by issuing the following command:

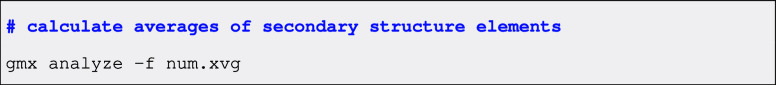



The resulting averages and associated error bars are listed in Table 1.

**Table 1:** Secondary Structures Assigned by the DSSP Algorithm^a^

Structure	Residue Count
Loops	16 ± 2
Bends	4 ± 2
Turns	13 ± 3
PPII-Helices	2 ± 2
π-Helices	0 ± 0
3_10_-Helices	2 ± 2
α-Helices	13 ± 2
β-Strands	23 ± 1
β-Bridges	2.1 ± 0.3

a Listed as the average number of residues of each type. The error bars are the “standard deviation” produced by analyze but given the strongly correlated nature of frames saved at 10-ps intervals, are more correctly referred to as a root-mean-square fluctuation.

The last analysis that will be demonstrated here is principal component analysis (PCA), a dimensionality reduction technique that in MD simulations is frequently used to determine low-frequency (fundamental) motions in biomolecules. The positional covariance matrix of a selection of atoms is computed, and the matrix is subsequently diagonalized to determine the characteristic eigenvectors and their associated eigenvalues. These eigenvectors can be used to visualize the slow motions in biomolecules. The GROMACS covar command computes the covariance matrix; in this example, we will choose the backbone atoms (group 4, “Backbone”) rather than including flexible side chains. The matrix is subsequently diagonalized by the anaeig command, which produces various outputs, such as the coordinates of extreme positions along each eigenvector and a projection of each trajectory frame along the selected principal components (here, the first two, as specified by -first 1 -last 2).

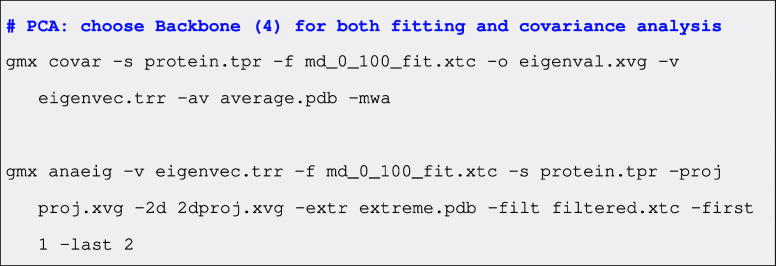



**Figure 6:**
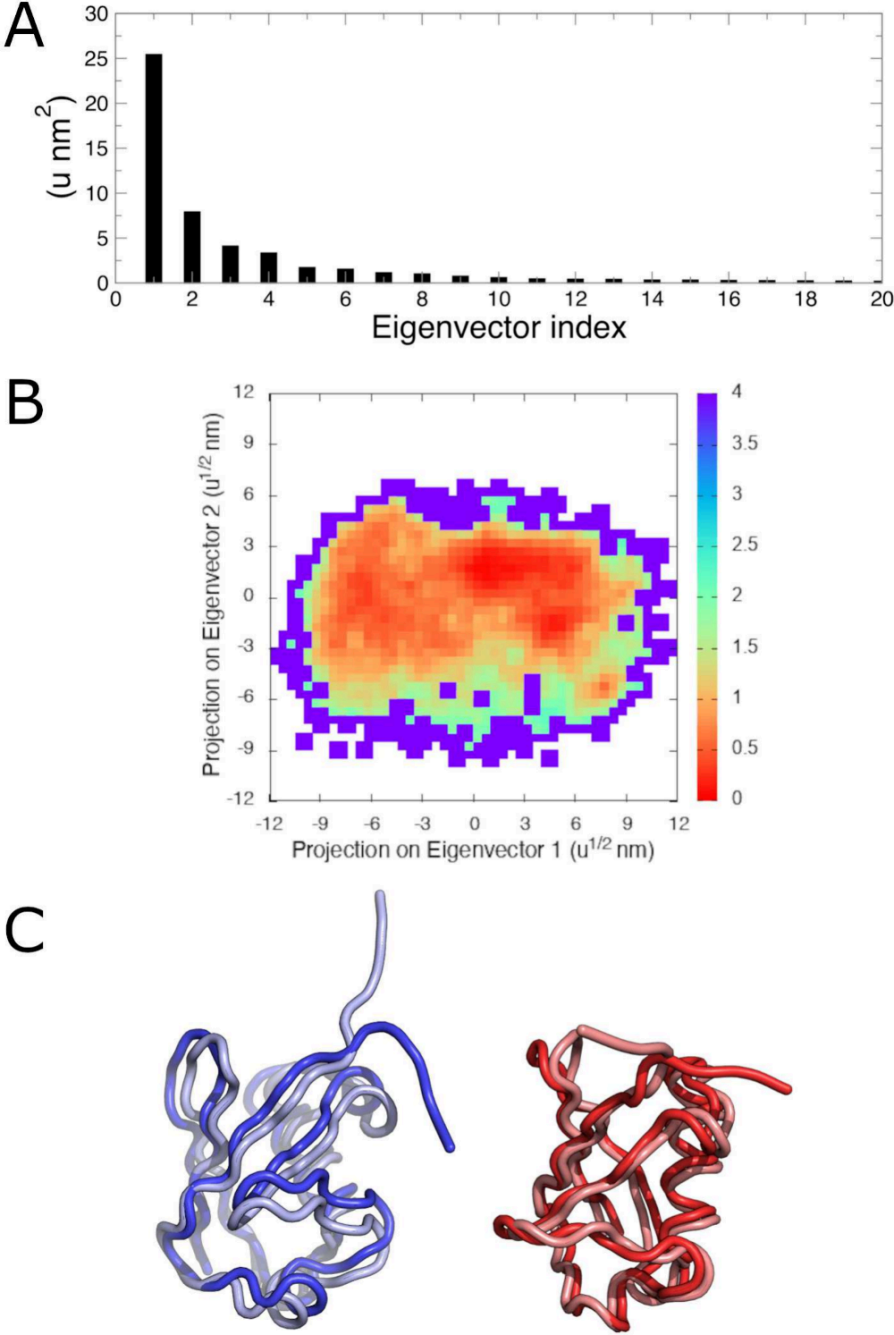
Principal component analysis of ubiquitin. (A) Eigenvalues for each of the first 20 eigenvectors. (B) Free energy landscape of all trajectory frames projected onto the first two principal components. The color bar corresponds to relative free energy in units of kcal mol^–1^. Relative free energies were computed as Δ*G* = −*k_B_T* ln[*P_i_*/*P*_*max*_], where *P*_*i*_ is the probability in a given bin and *P*_*max*_ is the probability in the bin of maximum occupancy. The two-dimensional histogram was generated over a range of {−12,12} with a bin width of 0.5 u^1/2^·nm in both dimensions. (C) Extreme structures along eigenvector 1 (blue) and eigenvector 2 (red).

Analysis of the covariance matrix produces 3*N* eigenvectors, where *N* is the number of atoms analyzed. In this case, from the 228 backbone atoms, 684 eigenvectors will be produced. Many of these eigenvectors will have very small magnitudes, therefore contributing little to the dynamics of the protein. As shown in Figure 6A, the first eigenvector has a much larger magnitude than all subsequent eigenvectors. It is for this reason that the subsequent projection of trajectory frames and extraction of extreme coordinates along each of these eigenvectors was limited to only the first and second eigenvectors when executing anaeig.

The trajectory frames are mapped onto the first two principal components to give an impression of free energy barriers between different conformations. This free energy surface is shown in Figure 6B. For this analysis, a two-dimensional histogram of the eigenvalues along the first two principal components was constructed using a bin width of 0.5 u^1/2^·nm. The resulting histogram was subsequently Boltzmann weighted and offset to zero at the bin of maximal occupancy to produce the free energy surface.

According to this analysis, the dominant motions in ubiquitin reside in the disordered C-terminal region. Principal component 1 corresponds to the C-terminal region swinging in one plane, and principal component 2 corresponds to a twisting in a plane orthogonal to the first (Figure 6C). This finding reinforces the flexibility indicated by RMSF analysis (Figure 4B).

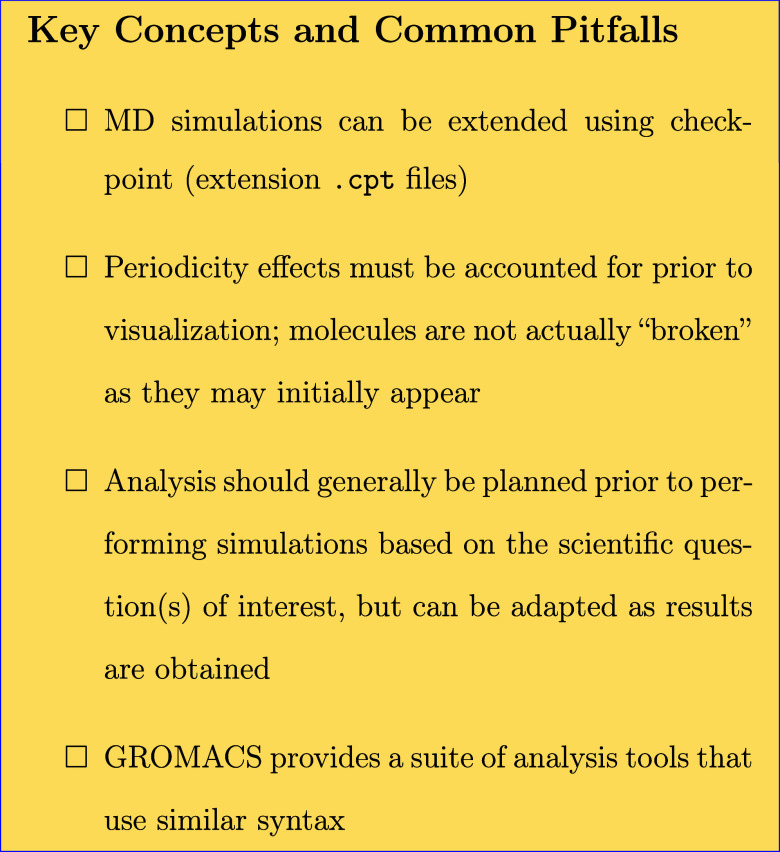



## Concluding Remarks

In this exercise, we have prepared a simulation system of the protein ubiquitin in an aqueous solvent, with mobile ions, performed equilibration and unrestrained MD, and analyzed the simulation outcomes. The general method shown here is applicable to any similar system of a biomolecule in water, but should not be viewed as the only way to set up, equilibrate, or analyze the system. We did not consider the important issue of convergence, that is, deciding how many replicate simulations to perform and when to terminate each simulation after having achieved adequate sampling. Convergence is a critically important consideration but is well beyond the scope of the tutorial presented here. Performing replicate simulations is also considered compulsory as any given trajectory may be a statistical outlier rather than a true indicator of equilibrium behavior and many independent simulations may be necessary.^41^ Additionally, the analysis presented here showcases the routine tasks one may wish to accomplish with GROMACS and should not be viewed as a recipe that needs to be, or even should be, followed. Indeed, we did not define true scientific questions or hypotheses here; it is these considerations that should motivate the analysis of MD trajectories. The workflow given here should provide a reasonable starting point for learning core GROMACS functions and performing simulations of a single biomolecule in water.

### Exercise 2: Protein Dimer in Water

In this exercise, we will prepare a simulation system that is a protein dimer. We will use the N-terminal PDZ domain of InaD in complex with a peptide from the C-terminal region of NorpA, taken from PDB: 1IHJ.^42^ As in Exercise 1, download the coordinates in PDB format from https://www.rcsb.org/structure/1IHJ as a starting point.

**Figure 7:**
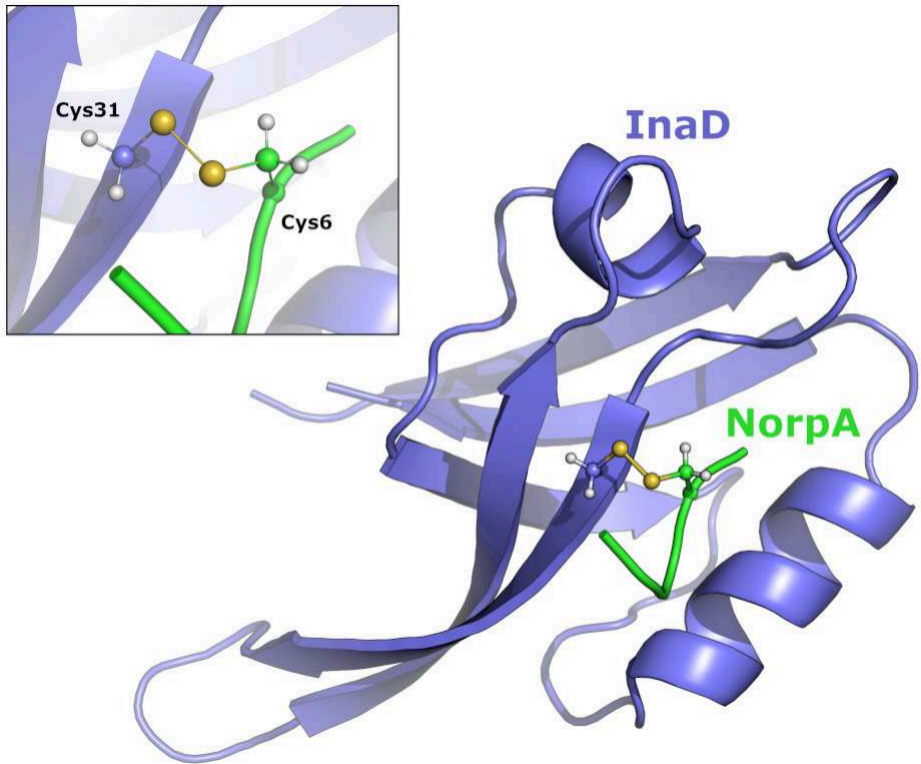
Structure of the InaD:NorpA complex, with InaD colored in blue and NorpA in green. The inset shows the detail of the intermolecular disulfide linkage between Cys31 of InaD and Cys6 of NorpA.

This system presents several additional levels of complexity compared to the ubiquitin system. First, the PDB file contains four polypeptide chains, because the InaD:NorpA complex crystallizes as a dimer of dimers. Therefore, we must separate out one dimer (either chain A/D or chain B/C) for the simulation. Second, the N- and C-termini of the InaD PDZ domain and the N-terminus of the NorpA peptide are missing residues, so we will have to carefully consider how to treat them. Third, the complex involves a disulfide linkage between the two polypeptide chains (Figure 7). Fourth, there are missing side chain atoms in several of the residues.

The fact that the protein complex contains multiple chains is no problem for the pdb2gmx program. Provided that each chain is assigned a unique chain identifier (letter or number) or is separated by a TER card, pdb2gmx can easily generate a topology for any complex of biomolecules. We must ensure that any input coordinate file provided to it meets these requirements.

#### Prepare the Structure

The first step in preparing the InaD:NorpA dimer for simulation is to extract two chains from the PDB file. For this example, we will extract the dimer that is formed by chains A and D. Use standard Linux commands grep and echo as shown below to isolate these chains.

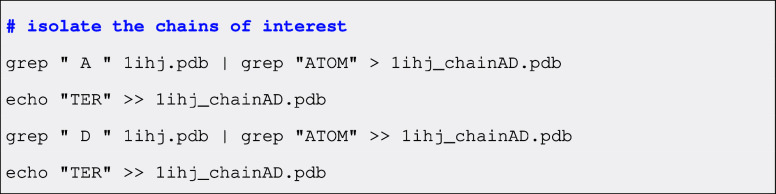



The crystal structure of InaD:NorpA is missing several terminal residues and a few side chain atoms. These missing entities will need to be modeled in. Rather than model in disordered terminal residues, which are unlikely to impact the dynamics in any functional way, it is commonplace to instead add “capping groups.” That is, rather than model the N- and C-termini in their ionized forms, which would be artificial in the case of incomplete chains, neutral groups can be added to mimic the presence of peptide bonds. N-termini are typically capped with acetyl groups and C-termini are typically capped with amide or *N*-methyl amide groups. Here, chain A (corresponding to InaD) is missing residues at both termini and chain D (corresponding to NorPA) is missing residues only at its N-terminus; its C-terminus is the true end of the polypeptide chain and therefore will not be altered.

The crystal structure is also missing several side chain atoms, in residues Asp95 and Phe105. It is common for flexible regions of a protein to have poor electron density in regions with mobile side chains. We will use the PyMOL Mutagenesis Wizard to reconstruct the missing atoms, and the residue building tool to add appropriate capping groups.

To reconstruct missing atoms in Asp95 and Phe105, open the Mutagenesis Wizard in PyMOL (from the top-level menu, Wizard → Mutagenesis → Protein). Click anywhere on Asp95 to select it, and from the right-hand menu, click “No Mutation” and toggle it to ASP... and choose ASP to correspond to the deprotonated (anionic) form of Asp. Repeat this procedure for Phe105 to build in its missing side chain atoms.

Next, add the capping groups. While this task can be completed with the Mutagenesis Wizard, it will attempt to uniformly cap all chains with the same entitites, which is not what is desired here. Instead, use the residue building feature. From the top-level menu, select Build → Residue → Acetyl. Doing so places an acetyl group in the middle of the viewing area. This group must be translated to a position that is suitable for connecting to the N-terminal residue of InaD (in this case, Gly12). Issue the following command in the PyMOL command prompt to move the acetyl group appropriately and merge its atoms into the chain definition of InaD.

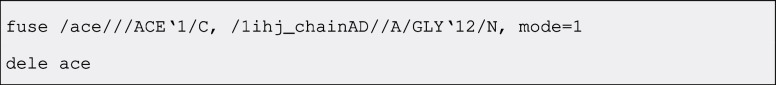



Perform a similar process for the C-terminus of InaD. From the top-level menu, select Build → Residue → *N*-Methyl. Move this group to a suitable position for connection to Phe105 with the following command.

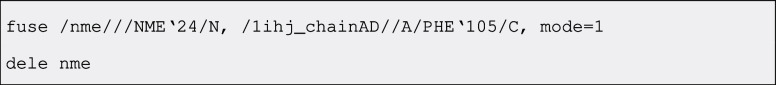



Repeat the process of adding an acetyl group to the N-terminus of NorpA. Save the modified coordinates in PDB format with File → Save Molecule → PDB format (name 1ihj_chainAD_capped.pdb).

The last step of preparation is to modify the added atom names to conform to the expectations of the force field. Atoms added to the amino acid side chains of Asp95 and Phe105 will be correctly named. The acetyl and *N*-methyl groups, however, require modification. Not only do the atoms have to be renamed, but they must appear as their own residues, named ACE and NME, to be properly processed by pdb2gmx. Open the PDB file obtained from PyMOL in a plain-text editor (like Vim or Emacs) and examine the atoms that have been added at the N-terminus of InaD, which will look something like the following:

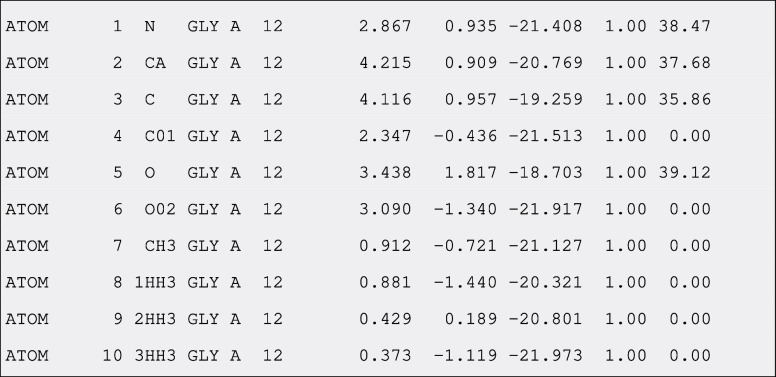



Rename these atoms to conform to the requirements of the force field definition, found in aminoacids.rtp in the charmm36.ff directory (C01 becomes C and O02 becomes O), rename the residue to ACE, and change the residue number from 12 to 11. When finished, the new N-terminus of the InaD protein (chain A) should look like the following:

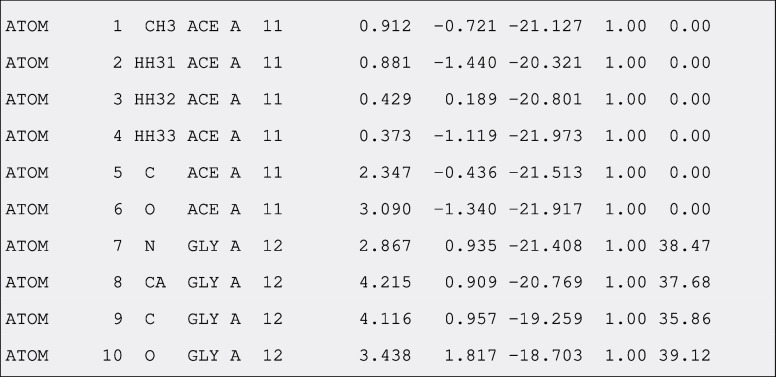



Similar adjustments are needed at the C-terminus of InaD to account for the *N*-methyl amide cap, which is called NME in the GROMACS implementation of the force field. When finished, the C-terminal residues of InaD should look like the following:

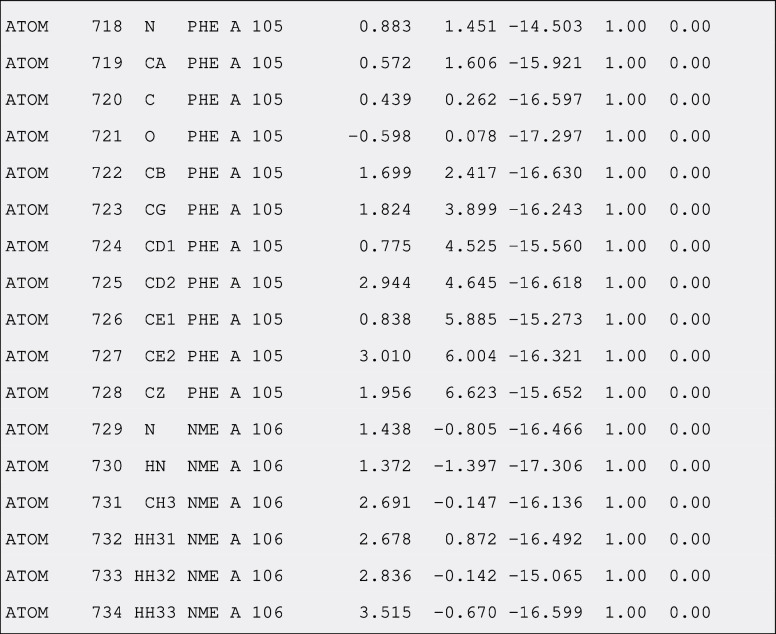



Repeat the procedure for fixing the acetyl cap at the N-terminus of NorpA, which is contained in chain D.

#### Write the Topology and Build the System

At this point, proceeding with preparing the simulation system is almost identical to that of ubiquitin in Exercise 1. In fact, pdb2gmx can handle any number of biomolecule chains, and protein complexes therefore require no special consideration in many cases. Only a few modifications are necessary to the procedure detailed above for the InaD:NorpA dimer system. Because an intermolecular disulfide linkage is formed between the two proteins, they must be considered in the same topological molecule definition. This convention does not mean the two proteins have to be one molecule in a chemical sense, rather their topologies have to be unified so the proper bonded interactions can be written to reflect the disulfide. Doing so is handled with the -merge option of pdb2gmx. The user can interactively merge specific chains, or choose to have all chains automatically merged into one topological molecule definition (called a “molecule type” in GROMACS).

Invoke pdb2gmx as follows, making the same selections as above for the force field and water model. The command specifies interactive selection of terminal patching via -ter, which is essential here. Without this option, pdb2gmx will attempt to treat the termini in their free amino and carboxylic acid forms, which will lead to an error because no such patching should be applied to capping groups. When prompted, choose None for the N-termini of both chains and the C-terminus of InaD. Choose COO- for the C-terminus of NorpA.

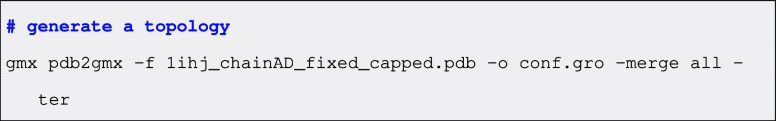



The terminal output of pdb2gmx includes an important distance matrix that is printed in all cases. We use it here as an illustrative example of the disulfide linkage that is present in this structure. The Sγ atoms of Cys31 in InaD and Cys6 in NorpA are at a distance of 0.204 nm. GROMACS uses a database file called specbond.dat that defines “special bonds” such as disulfides and linkages to prosthetic groups like heme. The reference distance for a disulfide bond is 0.2 nm, and GROMACS uses a 10% tolerance for all distances of this type. Therefore, any two Cys Sγ atoms that are within 0.18–0.22 nm apart will be assigned as being in a disulfide linkage unless overridden by the user via the -ss option, which prompts interactive assignment of cysteine oxidation states.

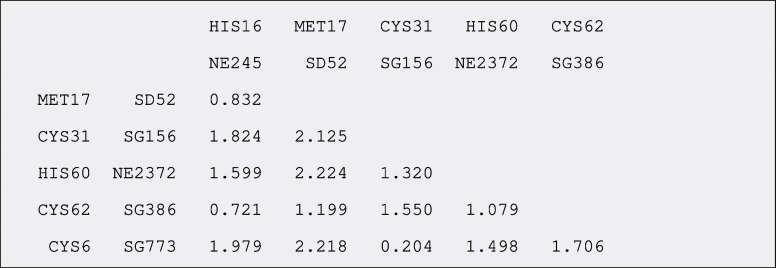



At this point, the protocol for constructing the system and performing an MD simulation is essentially the same as in Exercise 1, with one small difference. Proceed with defining a unit cell, filling it with water, and adding ions:

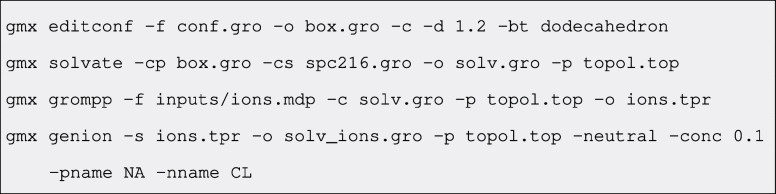



The only difference here is the use of the -neutral option when running genion to add 100 mM NaCl. This option is necessary because the InaD:NorpA complex carries a net −1 charge, unlike ubiquitin, which was electrically neutral. Therefore, genion will add one more Na^+^ ion than the number of Cl^–^ ions within the requested 100 mM NaCl to neutralize the charge of the system. The remaining protocol is identical as in the case of ubiquitin in Exercise 1, and therefore will not be repeated here.

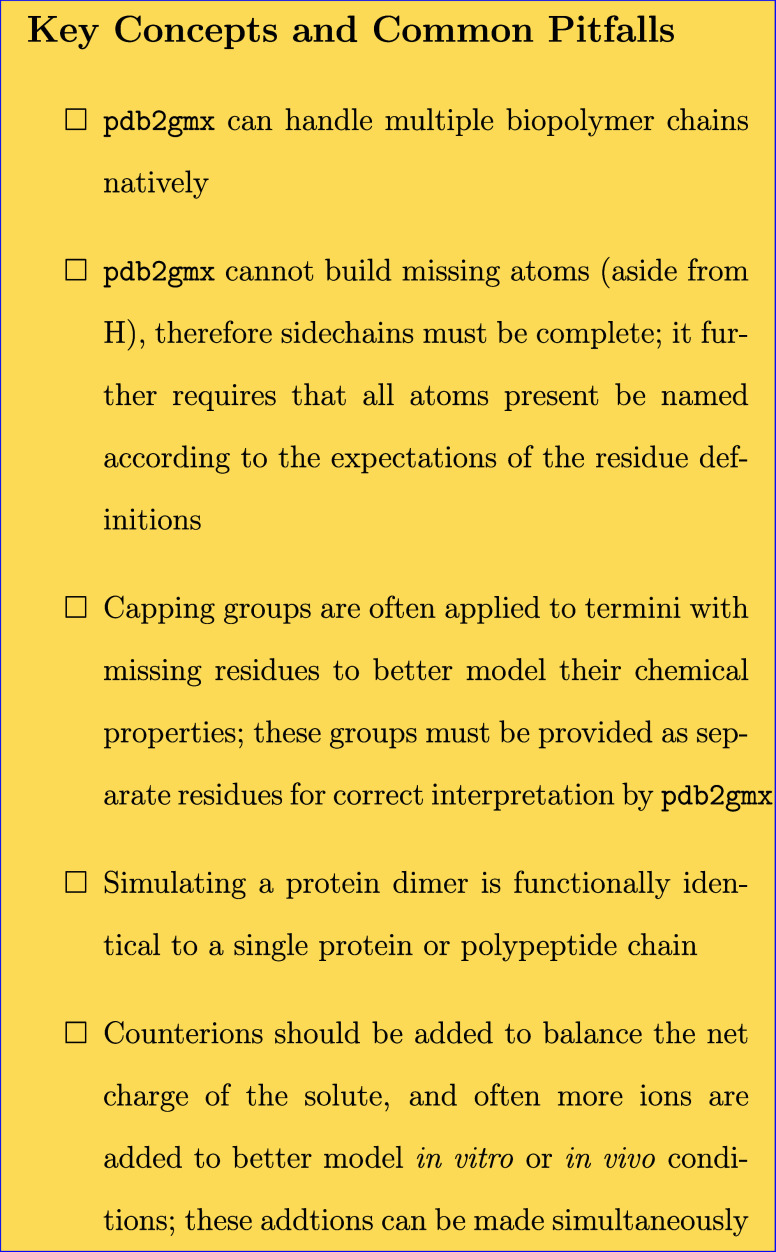



### Exercise 3: β-Hairpin Unfolding

#### Introduction to Umbrella Sampling

Unbiased MD simulations may not fully sample the configurational space for any system of interest, rendering it impractical, if not impossible, to calculate free energy differences between states. A central problem is the sparse sampling of high-energy states defining barriers between configurations, or the complete lack thereof. One strategy that has been developed to overcome this limitation is called umbrella sampling.^43^ In this method, a reaction coordinate, ξ, is defined (e.g., a distance or angle) and discrete regions along ξ are simulated. These regions are referred to as “windows” and a biasing potential is applied during MD simulations to restrict sampling to a narrow region of space around a target value at the center of each window.

Given the need to sample several windows along the reaction coordinate, umbrella sampling relies on independent MD simulations at each target value. Subsequent analysis is required to extract the potential of mean force (PMF)^44^ or a free energy surface that approximates the PMF. A common method for postprocessing umbrella sampling data is the Weighted Histogram Analysis Method (WHAM),^45^ which was implemented in GROMACS in 2010.^46^ The WHAM method discretizes the reaction coordinate to construct histograms of forces or coordinates in each window, which can subsequently be used to extract the unbiased probability distributions and the free energy values. Given that there are two unknowns, the unbiased probability distributions and the free energies, WHAM solves a system of equations iteratively. Interested readers are directed to an excellent article by Roux on this method and the relationship between umbrella sampling simulations and key concepts in statistical mechanics.^47^

#### Build the System

**Figure 8:**
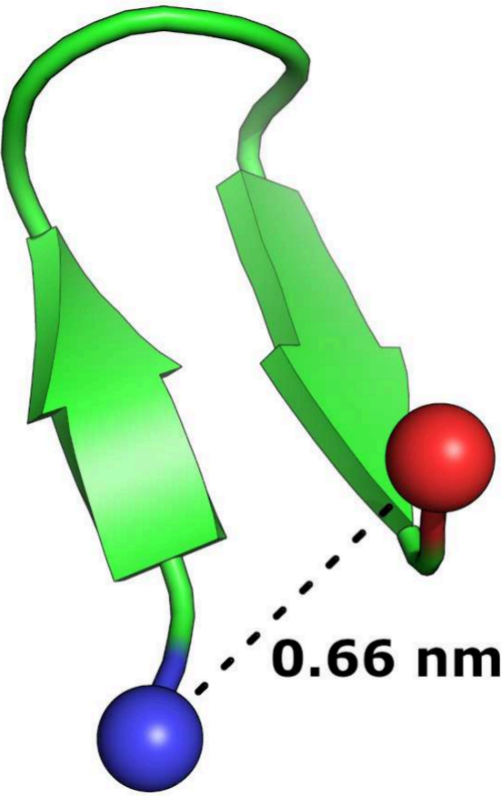
Structure of chignolin, from PDB: 1UAO. The reaction coordinate for umbrella sampling is indicated by the dashed line between the Cα atom of Gly1 and the Cα atom of Gly10, shown as blue and red spheres, respectively.

For this exercise, we will use the structure of chignolin (PDB: 1UAO, Figure 8).^48^ Download the coordinates in PDB format from https://www.rcsb.org/structure/1UAO. This PDB entry contains multiple models from an NMR ensemble, so we will extract the coordinates of the first deposited model with the Linux head utility:

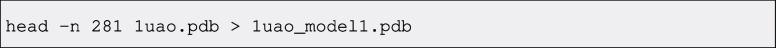



As with any other system, the first step in preparing the simulation system is to generate a topology. This task is performed as before with pdb2gmx. Note here the use of the -ignh option. In some cases, deposited structures do not adhere to typical nomenclature for hydrogen atoms, causing pdb2gmx to fail. A convenient solution is to ignore the presence of any hydrogen atoms (via -ignh) and have pdb2gmx rebuild them according to standard geometry rules for different functional groups. The nonstandard nomenclature for some hydrogen atoms in the chignolin structure prompts the use of this option. Otherwise, the hydrogen atoms have each be renamed in accordance with the expectations of the force field, which can be a tedious and error-prone task.

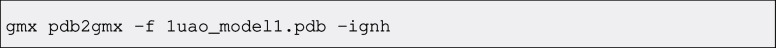



A key consideration in preparing the unit cell for a system that will be subjected to umbrella sampling is the length of the reaction coordinate that will be sampled. Each configuration that is simulated has to satisfy the minimum image convention, and the box must be sufficiently large such that the reaction coordinate length is less than half of the shortest box vector. This last requirement is due to the need to unambiguously define a distance between the two groups that define the reaction coordinate, rather than attempt to distinguish between distances within the central image and across periodic boundaries. In this case, we will be following a similar protocol as Sumi and Koga,^49^ sampling along a reaction coordinate with a maximum value of 3.0 nm.

For efficiency and to reflect the intrinsic (spherical) symmetry of the solute as it extends along the reaction coordinate, we will again employ a rhombic dodecahedral box shape. We will set a periodic image distance of 7.0 nm, which should remain at least twice as long as the longest reaction coordinate value and be sufficiently large to accommodate the protein without violating the minimum image convention. This value is set with the -box option.

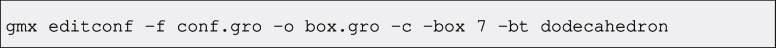



Solvating the unit cell proceeds as normal, with no special considerations.

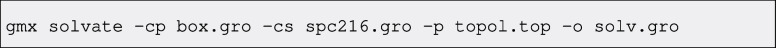



Here, we demonstrate the addition of neutralizing counterions with genion. In Exercise 2, we combined the -neutral option with a specified total concentration of mobile ions. Here, we use the -neutral option by itself to demonstrate that it can function without adding more ions beyond what are needed to neutralize the solute charge. In principle, one could specify any desired ionic strength here.

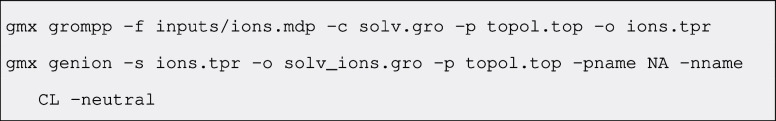



Minimization and equilibration are carried out as in the previous exercises.

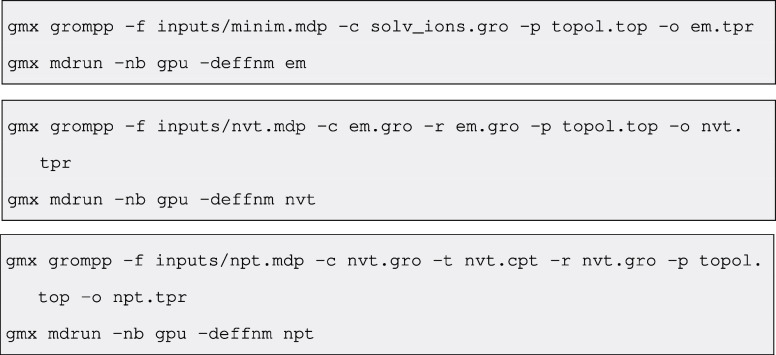



#### Generate Windows

The next step in the protocol is to generate the initial configurations needed to define the sampling windows along the chosen reaction coordinate. In the case of a linear reaction coordinate, such as the Cα-Cα distance we are using here, generating these configurations can be done by applying a biasing potential along this reaction coordinate to induce separation. That is, we will start with the native state in the PDB file (now minimized and equilibrated) and force the polypeptide to elongate along this vector.

To apply this bias, we need to define the two groups that will define the ends of the reaction coordinate, namely the Cα atoms of Gly1 and Gly10. Here, we introduce the concept of “index groups,” which GROMACS tools generically use for various input settings and analysis. The program best suited to generating static index groups is called make_ndx, which reads in a coordinate or binary topology file and prompts the user to make selections of atoms. Typing “help” at the make_ndx prompt gives examples and general syntax for making selections.

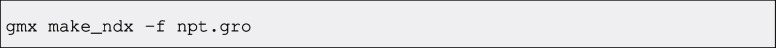



Here, we will be selecting one atom from each of two residues, assigning each atom to a group. We will also rename the groups for convenience. Below is the make_ndx prompt (indicated by the > sign) and the syntax for these selections and renaming operations. The r token indicates the residue being selected, a indicates the atom name, and the use of & signifies the union of these selections (the overlap, i.e. residues that satisfy both selections).

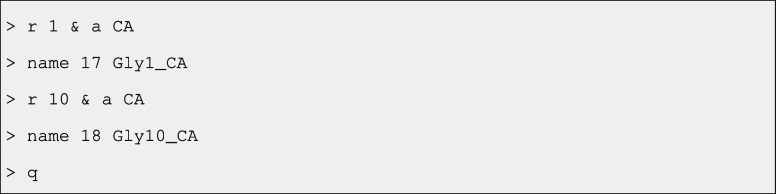



Having created these groups (which are written to a file called index.ndx by default), we can use them to apply a biasing force between them to generate initial configurations. The input .mdp file settings are shown below.

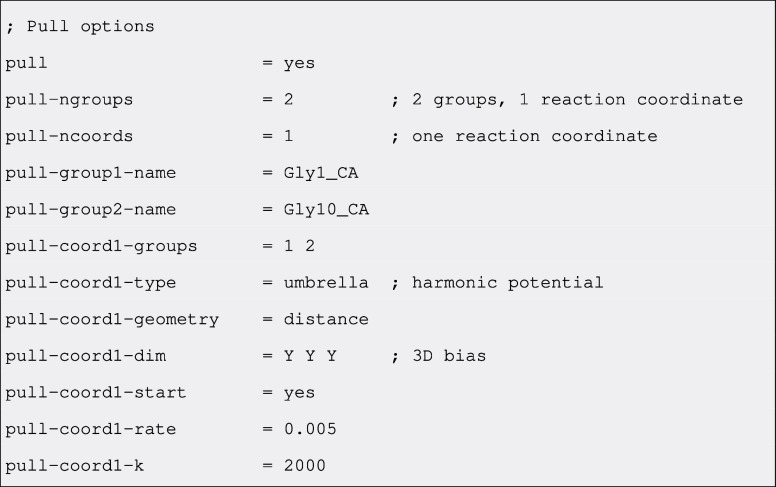



We are specifying that two groups (pull-ngroups = 2) will be used in total to define one pulling coordinate (pull-ncoords = 1). It is possible to define multiple biases simultaneously, among any arbitrary number of groups, although here we will use only one bias. The vector along which the bias is defined is specified by the names of the pulling groups (pull-group1-name and pull-group2-name) and assigning them to a coordinate (pull-coord1-groups = 1 2). We will apply a harmonic biasing force (also called an “umbrella potential”) via pull-coord1-type = umbrella that acts along all three Cartesian dimensions of the vector defining the distance between the two selected Cα atoms (pull-coord1-geometry = distance and pull-coord1-dim = Y Y Y). We will use the starting distance between the two atoms as the initial value for the pulling with pull-coord1-start = yes, but we will set this option differently for umbrella sampling (see below). The biasing force is exerted via an imaginary spring connecting the groups, and it will be elongated at a rate of 0.005 nm ps^–1^ and a force constant of 2000 kJ mol^–1^ nm^–2^ (via pull-coord1-rate = 0.005 and pull-coord1-k = 2000, respectively).

Again, invoke grompp to build the binary run input file, this time also supplying the index file that contains the custom groups. Without these definitions, grompp will fail with an error related to undefined groups. Then, perform the pulling simulation with mdrun.

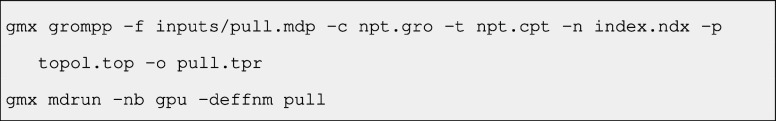



When the simulation is complete, measure the distance between the two Cα atoms over time with the distance command. This program allows for a selection of atoms that follows its own syntax that is more complex and flexible than that of make_ndx. For examples on the syntax for the distance command or any other GROMACS program that allows for dynamic selections, use gmx help selections.

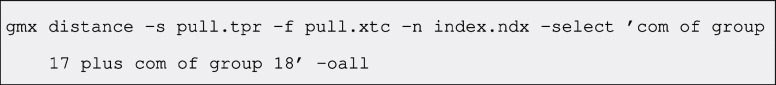



Each frame from the pulling simulation becomes a candidate for the starting structure of an umbrella sampling window. We will be sampling along the reaction coordinate from 0.5–3.0 nm, in intervals of 0.1 nm. Therefore, we need to identify snapshots that have Cα-Cα distances that are as close as possible to the desired values. This search can be scripted using any language of the user’s choosing, but here the following Python script is used:

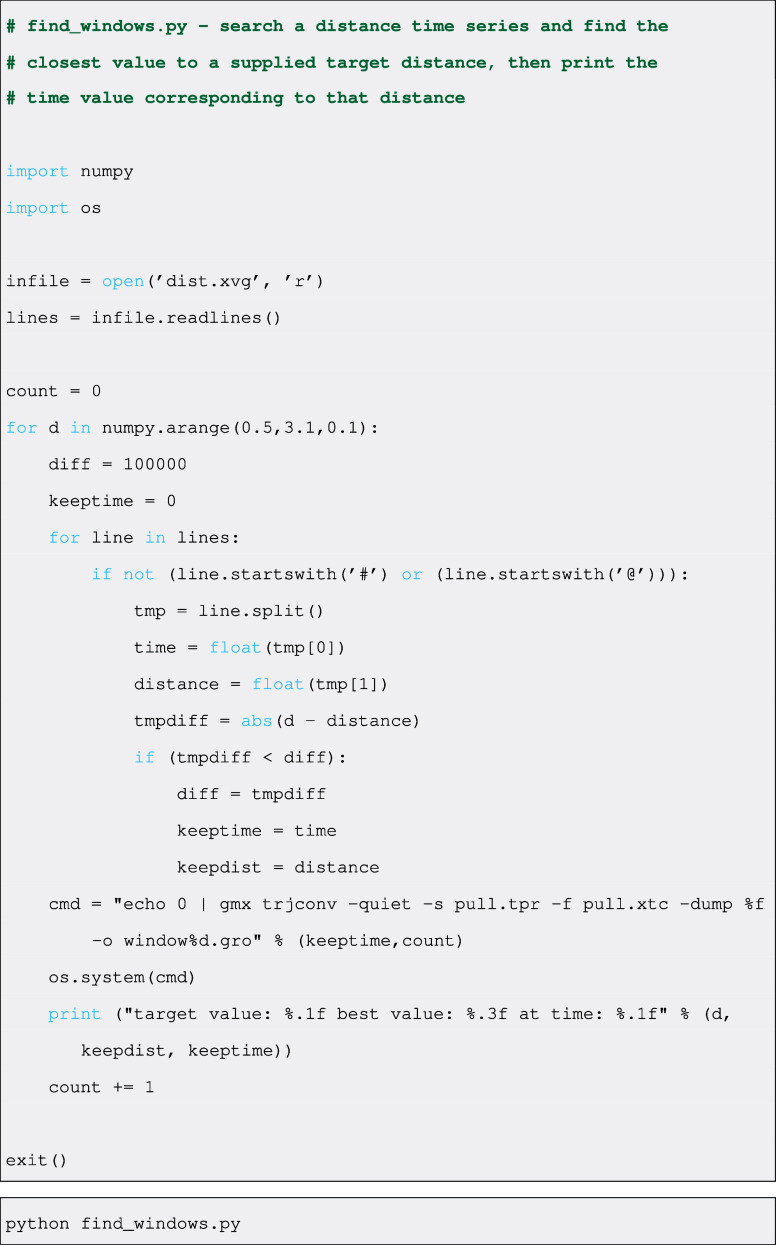



The script also calls the trjconv program to write out a coordinate file at the time that corresponds to the best match of the desired distance. These coordinates will subsequently be equilibrated and subjected to the umbrella sampling simulations.

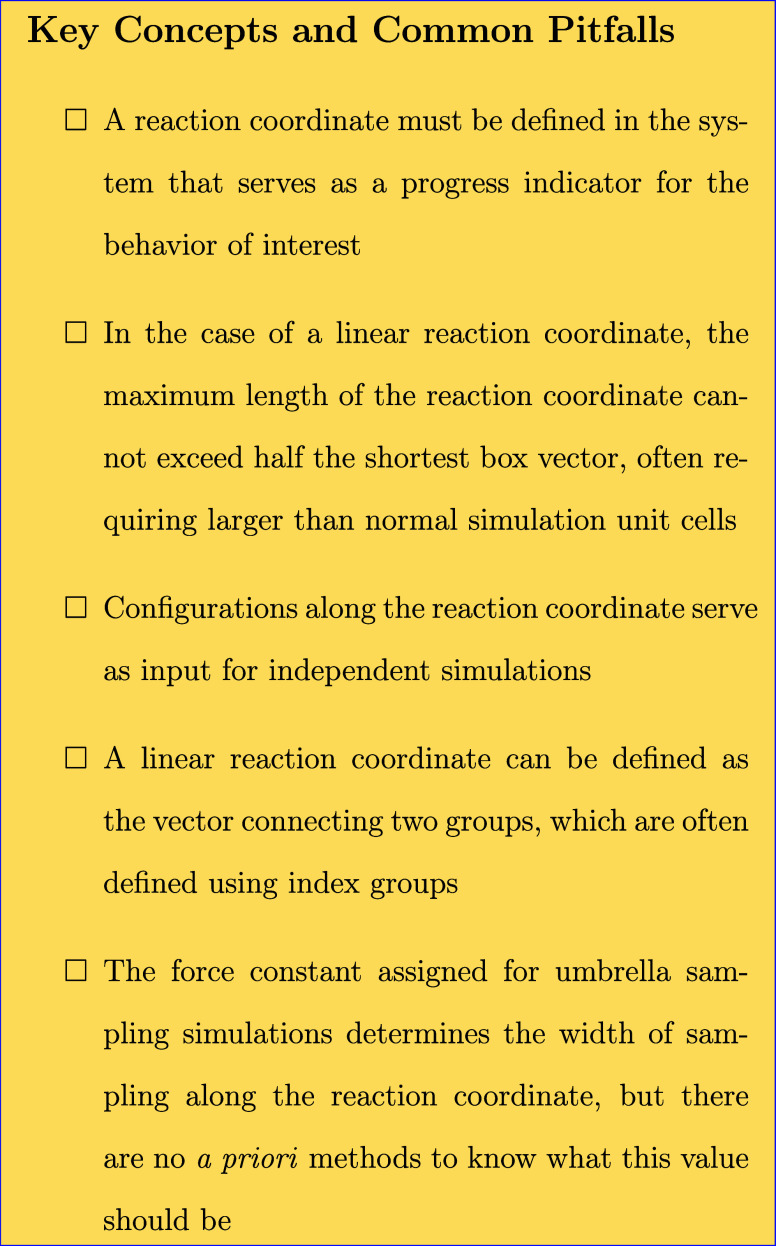



#### Perform Umbrella Sampling

A total of 26 windows define the desired reaction coordinate distance in the chignolin system. The approach shown here will run the simulations in a loop. There is no requirement to perform the simulations in this way, as each system can be simulated independently.

The .mdp settings that define the umbrella potential are nearly identical to those of the pulling simulation performed in the previous section. There are a few notable exceptions. Because we want to uniformly define 0.1 nm intervals for the umbrella windows, we will define these values explicitly. We will use a template file that has a replaceable field to generate real .mdp files; note the following settings that differ from the input settings shown above.

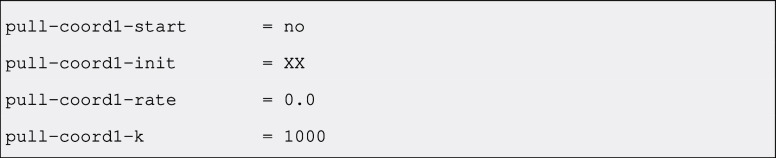



Execute the shell script below to replace the XX placeholder with real values. The use of pull-coord1-start = no tells grompp to ignore the initial distance between the defined groups. The reference distance for the biasing potential will thus be set to what is specified in pull-coord1-init, which will take regularly spaced values such as 0.5, 0.6, *etc*. This approach allows us to use coordinate files that are close, but not perfect, matches for the desired distances. For example, if in window 0, the reference distance between the Cα atoms is 0.507 nm, it can still be specified as being the reference for the desired value of 0.5 nm, allowing for uniform separation of window centers.

The value of pull-coord1-rate is set to zero, as we do not wish to apply any net force to the system to impart displacement. We only want to use the harmonic potential to restrain the distance around a set target. We also use a lower force constant here than what was applied during pulling; the magnitude of this force is somewhat of an empirical choice but as will be shown below, 1000 kJ mol^–1^ nm^–2^ yields good sampling. The larger value applied during window generation was necessary to induce the desired elongation in a practical amount of time.

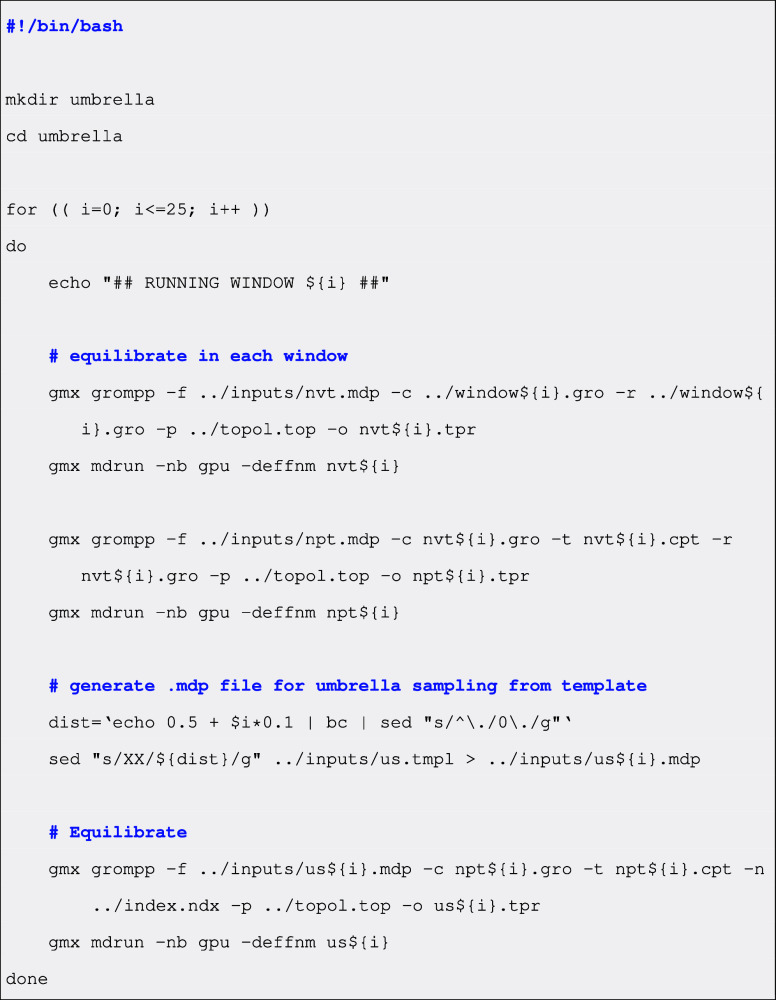



#### Analyze Results

The principal output from a series of umbrella sampling simulations is a free energy surface. In this case, we generate the profile from the WHAM algorithm.^45,46^ The wham program in GROMACS takes text files as input, each of which must specify the file names of the input .tpr files and either the output pull coordinate or pull force files. The program reads the biasing geometry and reference lengths from the .tpr files and the coordinate or force data from the outputs of mdrun to solve the WHAM equations. The input files can be prepared via a script like the following.

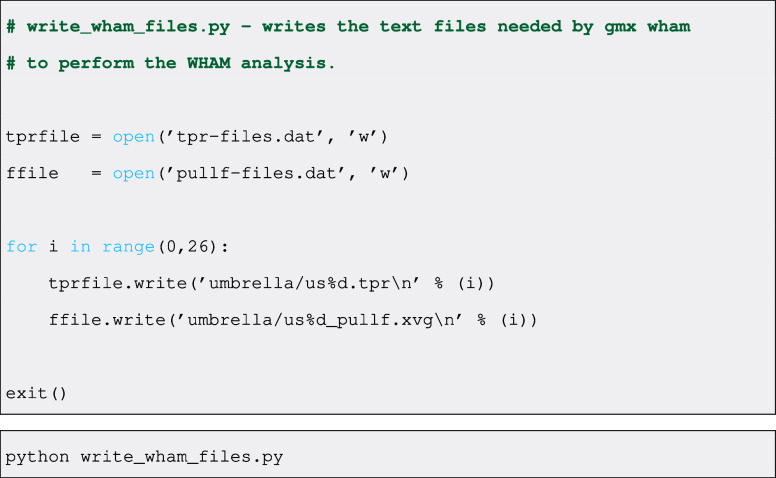



Once the output files are generated, execute the wham program.

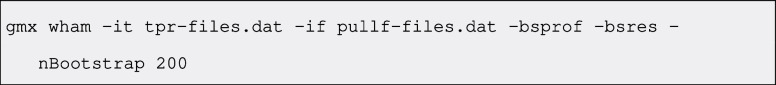



Note any messages from wham regarding areas of poor sampling. These warnings can indicate regions that will require additional sampling windows, an adjustment to window spacing, or changes to the force constant used. Here, we receive three warnings that there are windows with poor sampling at the extreme end of the defined reaction coordinate, and actually greater than the desired ending value of 3.0 nm.

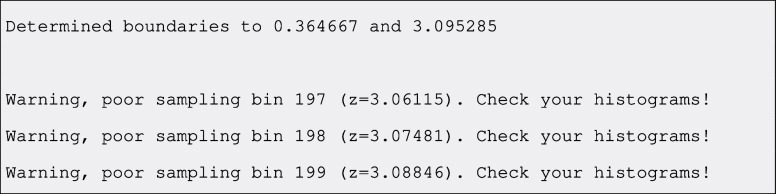



**Figure 9:**
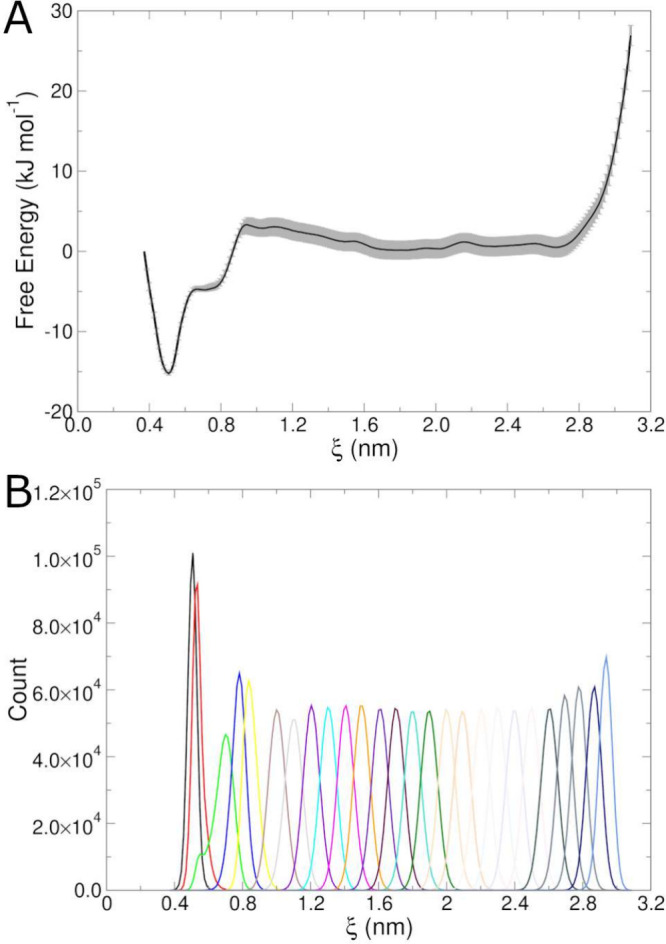
Umbrella sampling results for chignolin unfolding. (A) Free energy surface along the reaction coordinate, ξ, with gray shading indicating error bars from bootstrapping. (B) Histograms of sampling within each window along ξ.

The free energy surface generated by the WHAM algorithm is shown in Figure 9A. The global minimum corresponds to roughly 0.5-nm separation, which is expected based on the experimental structural ensemble. Error bars (shaded areas on the free energy surface) come from the default Bayesian bootstrapping algorithm built into the wham program, though they are likely an underestimate of the true error.^46^ The corresponding sampling histograms are shown in Figure 9B. The distributions are all overlapping to some extent, suggesting adequate coverage along the reaction coordinate. The sharp increase in free energy beyond ξ = 3.0 nm is a result of the few data points in that range, as the wham program warned. The chignolin peptide is fully elongated and somewhat strained at this distance, and these values are not likely to be physically meaningful. The overall shape of the curve, as well as the depth and position of the energy minimum, is similar to the result obtained in a previous study.^49^

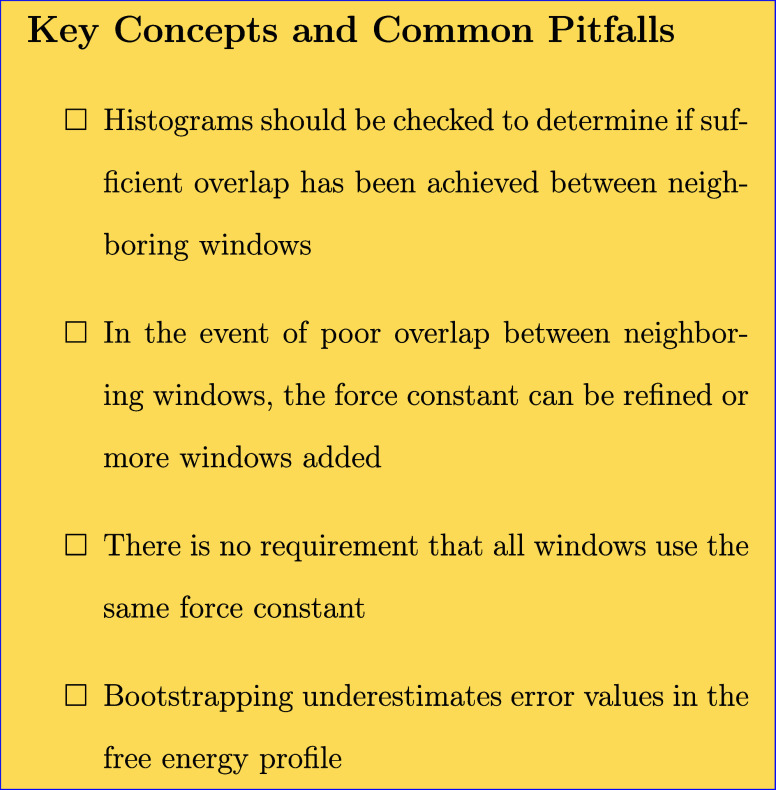



## Conclusions

Here, we have illustrated three basic workflows in the GROMACS simulation package. Exercise 1 detailed a routine procedure for simulating a globular protein in water. Exercise 2 expanded upon the first protocol and added intermolecular disulfide bonds and capping groups to the preparation steps and planning. Exercise 3 illustrated the use of a biasing potential to unfold a short polypeptide and calculate the free energy change associated with this process. The GROMACS software is capable of many more types of simulations and supports other enhanced sampling methods, but these core skills provide the foundation for other advanced applications.

## Data Availability

All input files necessary to reproduce the protocols here are provided via GitHub at https://github.com/Lemkul-Lab/gmx_tutorials_jpcb.

## Supporting Information Available

A compressed archive of all input files needed to reproduce the protocols described here (ZIP)
