# Supplementary figures and images for: Introductory Tutorials for Simulating Protein Dynamics with GROMACS
Source: J Phys Chem B. 2024 Sep 21;128(39):9418–35. doi: 10.1021/acs.jpcb.4c04901 (PMC11457149; doi:10.1021/acs.jpcb.4c04901)

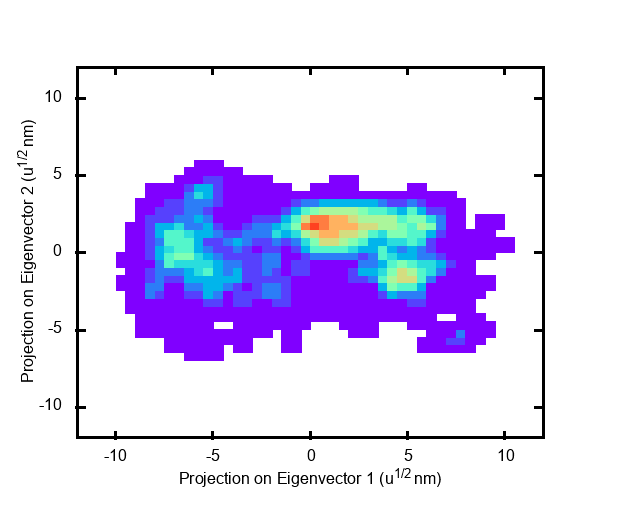

Supplement: Supplementary file 1 — jp4c04901_si_001.zip [file jp4c04901_si_001.zip › inputs/01_ubiquitin/free_ener_plot/norm_hist_d1_d2.png]

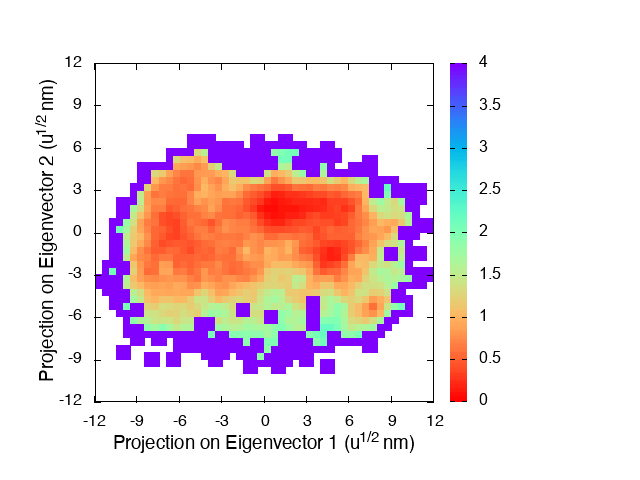

Supplement: Supplementary file 1 — jp4c04901_si_001.zip [file jp4c04901_si_001.zip › inputs/01_ubiquitin/free_ener_plot/free_ener_d1_d2.png]

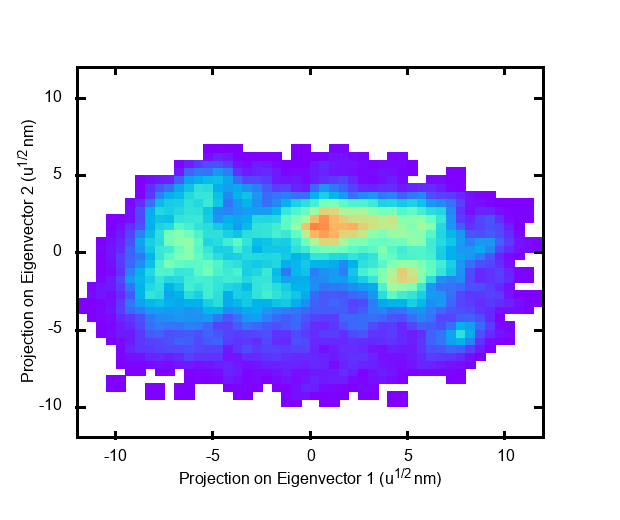

Supplement: Supplementary file 1 — jp4c04901_si_001.zip [file jp4c04901_si_001.zip › inputs/01_ubiquitin/free_ener_plot/hist_d1_d2.png]
